# Secrecy Performance of O-RAN-Enabled RIS-Assisted FSO/RF Satellite Downlinks

**DOI:** 10.3390/e28080907

**Published:** 2026-08-13

**Authors:** Yuhang Li, Xifan Chen, Jiale Shi, Guocheng Lv, Ye Jin

**Affiliations:** School of Electronics, Peking University, Beijing 100871, China; yuhangli@stu.pku.edu.cn (Y.L.); chenxifan@pku.edu.cn (X.C.); shijiale25@stu.pku.edu.cn (J.S.); jinye@pku.edu.cn (Y.J.)

**Keywords:** O-RAN, secrecy analysis, satellite-terrestrial communication, RIS-assisted communications, non-terrestrial networks

## Abstract

Motivated by the increasing security requirements of next-generation satellite-terrestrial communication systems and the emergence of Open Radio Access Network (O-RAN) architectures, this paper presents a secrecy analysis of a novel reconfigurable intelligent surface (RIS)-assisted mixed free-space optical (FSO) and radio frequency (RF) satellite downlink transmission system within an O-RAN-enabled non-terrestrial network (NTN) framework. The inherent broadcast nature of RF transmissions presents significant eavesdropping risks, which serves as the primary impetus for this study. We analyze the combined effects of imperfect channel state information (CSI) and random link blockage within such integrated networks. The impact of discrete phase shift constraints at the RIS is also investigated. Closed-form expressions are derived for three key performance metrics: connection outage probability (COP), secrecy outage probability (SOP), and the probability of positive secrecy capacity (PPSC). Through high signal-to-noise ratio (SNR) asymptotic analysis, corresponding asymptotic expressions are obtained, and all analytical results are validated via extensive Monte Carlo simulations. Our findings demonstrate that: (i) Link blockage probability and channel estimation accuracy jointly govern the secrecy performance floor. (ii) Increasing the number of RIS elements enhances physical-layer security by driving both the COP and SOP toward their theoretical lower bounds. (iii) Improving channel estimation accuracy diminishes the eavesdropper’s channel advantage and improves the overall system security. These results offer valuable insights for designing secure mixed FSO/RF satellite-terrestrial systems within O-RAN-enabled NTN architectures that effectively balance connectivity and confidentiality.

## 1. Introduction

The rapid expansion of satellite-terrestrial communication networks has revolutionized global data transmission. Geostationary Earth Orbit (GEO) satellites play a pivotal role in these networks by providing extensive coverage, with a single satellite capable of servicing nearly one-third of the Earth’s surface [1]. The integration of satellite communications (SatComs) into the Internet of Things (IoT) ecosystem is accelerating, driven by their capability to deliver extended coverage and enhanced data rates across diverse application scenarios [2]. These systems provide significant advantages, such as wide-area connectivity, high-capacity backhaul, and reliable service continuity, making them indispensable to modern communication infrastructure [3]. With the emergence of O-RAN architectures enabling open, multi-vendor interoperability across terrestrial and non-terrestrial segments, satellite-terrestrial networks are poised for even deeper integration into next-generation cellular systems. However, multipath effects, caused by signal scattering from ground receivers, introduce numerous non-line-of-sight (NLoS) components that create masking effects and disrupt line-of-sight (LoS) communications [4]. This issue necessitates deploying terrestrial relays to enhance communication quality and ensure comprehensive coverage [5]. Furthermore, the inherent openness of wireless propagation presents critical security vulnerabilities, as eavesdroppers can potentially intercept sensitive information [6]. The unique characteristics of satellite communication systems, including long propagation distances, high orbital dynamics, and broadcast downlink transmissions, further exacerbate these security concerns and have motivated extensive research on security solutions for satellite-terrestrial networks [7]. Within the O-RAN framework, the open fronthaul and disaggregated RAN functions create new attack surfaces that demand rigorous physical-layer security (PLS) guarantees. Consequently, it is imperative to develop robust solutions that ensure both reliable connectivity and information security in satellite-terrestrial systems.

In parallel, the Open Radio Access Network (O-RAN) paradigm is reshaping next-generation cellular and non-terrestrial network (NTN) architectures by promoting openness, intelligence, and multi-vendor interoperability through standardized open interfaces, cloud-native softwarization, and AI/ML-driven control loops [8]. The integration of O-RAN principles into satellite-terrestrial networks enables flexible, software-defined management of heterogeneous space–air–ground segments, facilitates AI-native orchestration for fast-varying channel dynamics, and supports virtualized deployment of RAN functions across terrestrial and non-terrestrial nodes. Within this evolving O-RAN-enabled NTN ecosystem, ensuring robust physical-layer security (PLS) against eavesdropping threats becomes both more critical and more challenging, as the open and disaggregated architecture expands the attack surface while simultaneously offering new opportunities for AI-driven security countermeasures.

Free-space optical (FSO) communication has emerged as a promising alternative to traditional radio frequency (RF)-based satellite systems, offering significant advantages such as ultra-high bandwidth, with demonstrated transmission rates exceeding 1.2 Tbps or 1200 Gbps [9], enhanced link security [10], immunity to electromagnetic interference (EMI) [11], and lower deployment costs [12]. The EMI immunity arises from the fact that FSO links operate at optical frequencies, far above the congested RF spectrum, making them inherently impervious to terrestrial RF interference sources, deliberate jamming attempts, and spectrum licensing constraints. In a typical FSO link, intensity modulation with direct detection (IM/DD) is employed, where the intensity of the optical carrier is modulated by the transmitted signal and the received optical power is directly converted to an electrical signal at the photodetector. When combined with RF relays, heterodyne detection (HD) is often adopted to mix the received optical signal with a local oscillator, thereby improving receiver sensitivity and enabling subcarrier-based retransmission over the RF link. However, FSO systems face fundamental limitations due to atmospheric turbulence, which induces beam scintillation and wander, as well as pointing errors caused by factors like building sway from thermal expansion, wind loads, and microseismic activity [13]. These effects can lead to frequent link outages and severe performance degradation. Meanwhile, although conventional RF satellite systems are widely deployed across various frequency bands, they suffer from inherent drawbacks, including spectrum congestion, security vulnerabilities, and inter-band interference [14,15].

To harness their complementary strengths, integrated FSO/RF architectures have been proposed as an optimal solution that synergistically combines both technologies. Among the various integration paradigms—mixed (dual-hop relaying), hybrid (parallel links with diversity), and switching (backup RF when FSO fails)—this work adopts the mixed FSO/RF architecture within an O-RAN-enabled NTN context, where the satellite-to-relay hop is an FSO link and the relay-to-user hop is a reconfigurable intelligent surface (RIS)-assisted RF downlink managed through O-RAN’s open control interfaces. By incorporating relay technology that performs photoelectric conversion between optical and RF domains, mixed FSO/RF systems harness the combined benefits of both communication technologies [15].

Modern satellite-terrestrial networks confront two fundamental challenges: RF transmissions suffer from multipath fading and terrain-induced degradation [16], while their omnidirectional nature creates systemic eavesdropping vulnerabilities [17]. Reconfigurable intelligent surface (RIS) technology addresses both challenges by dynamically optimizing reflected signal phases [18], enhancing coverage and capacity [19,20,21,22,23]. Within an O-RAN-enabled NTN architecture, RIS phase configurations can be controlled by xApps on the near-RT RIC, enabling closed-loop propagation optimization. RIS also serves as a countermeasure against jamming attacks [24] and can degrade eavesdroppers’ reception through randomized radiation [25]. These challenges are further compounded by imperfect CSI and random link blockage [26], which existing schemes rarely address jointly, creating a gap between theory and practice.

### 1.1. Related Works

Hybrid FSO/RF systems have received considerable attention for their potential to enhance both the reliability and capacity of IoT wireless communications. With the advent of O-RAN-enabled NTN architectures that demand rigorous security guarantees across open, multi-vendor interfaces, the need for a unified treatment of PLS in such systems has become increasingly urgent. While recent studies have explored various aspects of these systems, a unified treatment of their practical deployment constraints remains absent. As summarized in Table 1, early foundational works [27,28] established channel models for FSO/RF links using Gamma-Gamma, EG, Rayleigh, and $K_{G}$-fading distributions. However, these studies neither considered secrecy performance nor incorporated RIS assistance, and they relied on idealized assumptions of perfect CSI and unobstructed links. Subsequent physical-layer security (PLS) analyses in non-RIS-assisted systems [29,30,31] employed various fading models, including Nakagami-*m* and η-μ fading, to evaluate secrecy metrics. While these works advanced the understanding of mixed FSO/RF security, they maintained the perfect CSI assumption and did not account for either RIS integration or link blockage effects. Ref. [32] further investigated PLS for a parallel mixed FSO/RF system with multiple independent eavesdroppers on both FSO and RF links, considering both collusion and non-collusion scenarios, yet this work also omitted RIS assistance and blockage considerations. Ref. [33] extended the secrecy analysis to non-terrestrial network architectures by studying artificial noise (AN)-aided PLS for mixed FSO-RF systems, demonstrating the effectiveness of AN-based countermeasures in non-terrestrial networks (NTN). However, ref. [33] still assumed perfect CSI and unobstructed links, without incorporating RIS assistance. Notably, ref. [34] introduced RIS into satellite-terrestrial secrecy analysis for integrated satellite-vehicle networks. While [34] incorporated RIS assistance, it assumed perfect CSI and did not consider link blockage.

Despite these advances, a critical limitation persists across the existing literature: nearly all prior works address only a subset of the practical challenges that arise in real-world deployments. Specifically, no existing study simultaneously incorporates (i) RIS-assisted transmission, (ii) imperfect CSI, and (iii) random link blockage within a unified secrecy analysis framework for mixed FSO/RF satellite downlinks—a gap that is particularly consequential for O-RAN-enabled NTN deployments where security must be guaranteed across open, disaggregated network segments. The emergence of RIS-assisted networks has marked a significant evolution in wireless communications. For instance, ref. [35] combined RIS with secrecy-enabled mixed FSO/RF systems using Gamma-Gamma and Nakagami-*m* fading models. It is important to note that [35] focuses on an uplink heterogeneous network scenario where terrestrial users transmit to a base station via RIS-assisted RF links and an FSO backhaul, and assumes perfect CSI without considering link blockage. In contrast, our work addresses a satellite downlink scenario with a GEO satellite transmitting to terrestrial users through a relay, where the FSO link is subject to long-distance atmospheric turbulence and pointing errors, and the RF downlink is vulnerable to both imperfect CSI and random blockages. These architectural differences lead to fundamentally distinct channel characteristics and system design considerations. Similarly, ref. [36] introduced imperfect CSI to RIS-aided systems using the Málaga (M) distribution, and [37] advanced secrecy analysis under imperfect CSI for M-distributed FSO and Nakagami-*m* RF channels, yet neither of these works considered RIS assistance and link blockage simultaneously.

More recently, several studies have adopted heterogeneous RF channel models to better capture the physical propagation characteristics of different sub-links in RIS-assisted systems. For instance, ref. [38] considered a mixed FSO/active RIS-aided nonorthogonal multiple access (NOMA) system where the relay-to-RIS and RIS-to-user links are modeled by Rician fading to exploit the strong LoS components, while the direct relay-to-user link follows Rayleigh fading due to the absence of a dominant LoS path. Similar heterogeneous modeling approaches have been employed in [39,40,41], where the RIS-assisted cascaded links are characterized by Rician or Nakagami-*m* fading, while the direct links are modeled by Rayleigh fading. These heterogeneous models provide a more physically accurate representation of realistic deployment scenarios at the cost of increased analytical complexity. In contrast, our work adopts a homogeneous Rayleigh fading assumption for all RF sub-links. While this choice sacrifices some modeling fidelity, it enables the derivation of tractable closed-form expressions for key secrecy metrics under the combined effects of imperfect CSI and link blockage, which is a level of analytical tractability that is challenging to achieve with heterogeneous fading models. We note that extending the proposed secrecy analysis framework to incorporate heterogeneous RF channel models constitutes an important direction for future work, as it would allow quantification of the security benefits arising from the LoS advantage of RIS-reflected paths.

This work intersects several rapidly evolving research areas that have been the subject of recent comprehensive surveys, further underscoring its timeliness. In RIS-assisted PLS, Khoshafa et al. [42] provided a thorough review of PLS techniques in both RF and optical wireless systems, highlighting RIS as a key enabler for secure 6G communications. For satellite communication security, Abdelsalam et al. [43] surveyed PLS solutions across hybrid satellite-terrestrial, IoT, and FSO-based satellite systems, identifying the need for unified frameworks that account for practical impairments. In the O-RAN domain, Soltani et al. [44] examined the security implications of intelligent control in 6G Open RAN, discussing both the risks of open interfaces and the opportunities for AI/ML-driven security countermeasures.

From a channel estimation perspective, several advanced methods have been proposed to improve CSI acquisition accuracy in complex wireless environments. For instance, sparse Bayesian learning (SBL) techniques [45] exploit the inherent sparsity of wireless channels to achieve robust estimation performance even with limited pilot resources. Such methods are particularly relevant in RIS-assisted systems, where the cascaded channel exhibits structured sparsity that can be leveraged to enhance estimation accuracy. In the context of O-RAN-enabled NTN architectures, these advanced estimation algorithms can be implemented as xApps within the near-RT RIC, enabling adaptive pilot allocation and CSI refinement strategies that directly improve the CSI estimation accuracy in our analytical framework.

### 1.2. Motivations and Contributions

To address the identified gap, we propose a comprehensive analytical framework for RIS-assisted mixed FSO/RF satellite downlinks that jointly accounts for imperfect CSI, random link blockage, and finite-resolution RIS phase quantization. We adopt the M distribution for the FSO link, which unifies most existing statistical turbulence models (including Gamma-Gamma and log-normal) as special cases and shows excellent agreement with published data from weak to strong turbulence. The main contributions of this work are summarized as follows:

- Unified analytical framework for RIS-assisted mixed FSO/RF satellite downlink: We propose an RIS-assisted mixed FSO/RF satellite downlink transmission system and develop the unified analytical framework that jointly accounts for imperfect CSI, random link blockage, pointing errors, and M turbulence. Closed-form expressions for the cumulative distribution function (CDF) and the probability density function (PDF) of the end-to-end SNR are derived under both HD and IM/DD techniques.
- Comprehensive reliability and PLS analysis: We conduct a thorough performance analysis by deriving closed-form expressions for the connection outage probability (COP) as a reliability metric, and the secrecy outage probability (SOP) and the probability of positive secrecy capacity (PPSC) as the primary secrecy metrics. Asymptotic expressions are further obtained at high SNR. We prove that the presence of link blockage and imperfect CSI yields a secrecy diversity order of zero, indicating that the SOP saturates to a non-zero floor regardless of SNR.
- Practical RIS modeling with discrete phase shifts: In contrast to most prior works that assume ideal continuous RIS phase shifts, we recognize that practical RIS implementations employ finite-bit phase shifters due to hardware cost and power constraints. Quantifying the resulting secrecy performance degradation is critical for determining the minimum phase resolution required to meet given security targets. We extend the analysis to incorporate discrete phase shift constraints at the RIS, capturing the finite-resolution limitation of practical reflecting elements. The impact of phase quantization on the secrecy performance is quantified, and the performance gap relative to the ideal continuous phase shift case is evaluated through numerical results.
- Design guidelines for secure system deployment: Through extensive numerical analysis and the derived asymptotic expressions, we demonstrate how CSI estimation accuracy, RIS element count, blockage probability, and discrete phase shift resolution collectively govern the secrecy performance. A dedicated design insights subsection provides actionable guidelines for RIS configuration, CSI quality requirements, blockage mitigation strategies, and power allocation, bridging the gap between theoretical analysis and practical system deployment.

Our work provides concrete design guidelines for next-generation secure O-RAN-enabled satellite-terrestrial communication architectures, offering analytical tools that can be embedded within the O-RAN control framework to effectively balance connectivity with confidentiality requirements in open, multi-vendor NTN deployments.

### 1.3. Structure

The paper is structured as follows: Section 2 introduces the O-RAN-enabled RIS-assisted mixed FSO/RF satellite downlink transmission system model, and provides a detailed statistical characterization of the end-to-end SNR for both the FSO and RF links. Section 3 presents a thorough physical-layer security analysis under imperfect CSI and random link blockage, deriving analytical closed-form expressions for critical performance metrics such as the SOP, COP, and PPSC, which can serve as the analytical backbone for security-oriented xApps in the O-RAN RIC. In Section 4, Monte Carlo (MC) simulations are used to validate the derived analytical expressions, and several practical insights are discussed regarding the effects of various system parameters on secrecy performance. Finally, Section 5 concludes the paper by summarizing the main findings and highlighting potential directions for future research.

Notations: $\mathrm{CN}(0,\sigma^{2})$ denotes the circularly symmetric complex Gaussian (CSCG) distribution with zero mean and covariance matrix $\sigma^{2}$; ∼ stands for “distributed as”; $G_{p,q}^{m,n}\left[\cdot \right]$ is the Meijer-*G* function as defined in [46] (Equation (8.2)); δ(·) is the Dirac delta function [46]; Γ(·) represents the gamma function as defined in [46] (p. 759); P(·) represents the probability measure; $H_{p1,q1;p2,q2;p3,q3}^{0,n1;m2,n2;m3,n3}\left[\cdot \right]$ denotes the extended generalized bivariate *H* function that encapsulates the solution’s transcendental structure; $E\left[\cdot \right]$ denotes the statistical expectation.

## 2. System Model and Statistical Analysis

**Figure 1:**
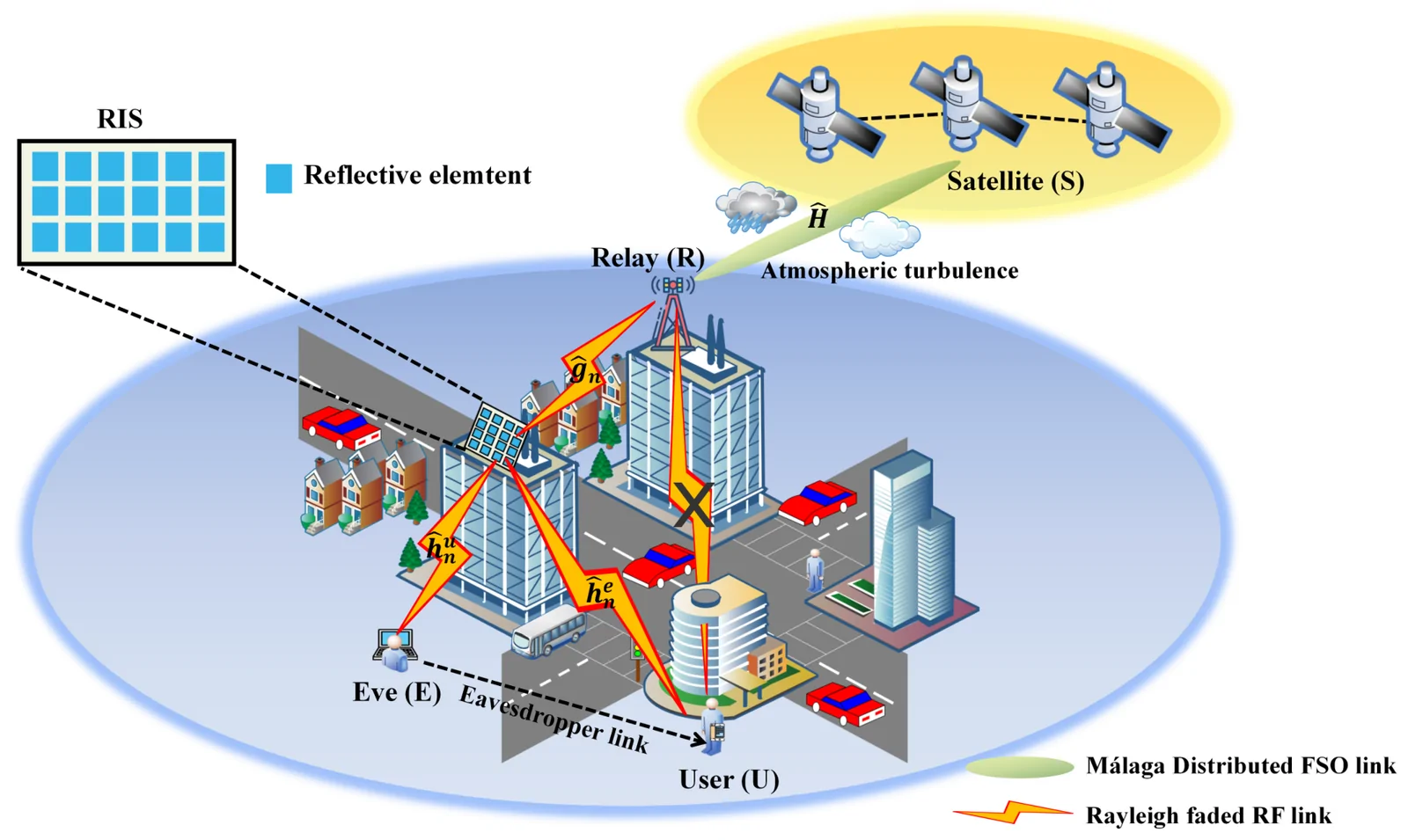
O-RAN-enabled RIS-assisted mixed FSO/RF satellite downlink transmission system.

We investigate an O-RAN-enabled RIS-assisted mixed FSO/RF satellite downlink system under imperfect CSI and random link blockages, as shown in Figure 1. A geostationary satellite (S) transmits optical signals to a terrestrial relay (R) operating in fixed-gain amplify-and-forward (AF) mode. The relay performs photoelectric conversion, amplifies the signal, and retransmits it as an RF signal to a legitimate user (U) [15]. The RIS operates as a passive reflector introducing only phase shifts, imposing no additional computational burden on ground terminals. An eavesdropper (E) attempts to intercept the RF transmission. The FSO link is modeled by the M distribution to characterize atmospheric turbulence, while Rayleigh fading captures multipath effects in the terrestrial RF environment.

### 2.1. FSO Link

The FSO link serves as the high-capacity backhaul from S to R. Channel coefficients are assumed constant per codeword block and independent across blocks [47,48]; satellite orbital dynamics are neglected for the quasi-static GEO platform [49]. The electrical signal received at R is

(1) $$\begin{array}{c} y_{1}=\sqrt{P_{1}}\eta Hs+n_{1}, \end{array}$$

where $P_{1}$, η, *s*, and $n_{1}∼\mathrm{CN}\left(0,\sigma_{1}^{2}\right)$ denote the transmit power of S, the optical-electrical conversion coefficient, the transmitted signal with unit energy, and the additive white Gaussian noise (AWGN) at R, respectively.

The composite FSO channel gain is given by $H=L_{1}h_{m}h_{a}h_{p}$ [35,36], whose constituent components are defined as follows: $h_{m}$ is the atmospheric turbulence fading coefficient; $h_{a}$ is the channel attenuation loss under specific weather conditions; and $h_{p}$ is the pointing error. The probability density function (PDF) of $h_{p}$ is given by [50]

(2) $$\begin{array}{c} f_{h_{p}}\left(h_{p}\right)=\frac{\psi^{2}}{A_{0}^{\psi^{2}}}h_{p}^{\psi^{2}-1},\;0<h_{p}\leq A_{0}, \end{array}$$

where $A_{0}$ and ψ denote the power collection coefficient and the ratio between the receive beam radius at the relay and the pointing jitter standard deviation, respectively.

The propagation loss $L_{1}$ of the FSO link is expressed as [51]

(3) $$\begin{array}{c} L_{1}=G_{a}G_{b}{\left[\frac{\lambda }{4\pi d_{s}}\left(\frac{J_{1}\left(\mu \right)}{2\mu }+36\frac{J_{3}\left(\mu \right)}{\mu^{3}}\right)\right]}^{2}, \end{array}$$

with the following parameter definitions: λ is the wavelength of the satellite optical carrier signal; $d_{s}$ is the distance of the S-R link; $G_{a}$ is the satellite beam gain; $J_{1}\left(\cdot \right)$ and $J_{3}\left(\cdot \right)$ are Bessel functions of the first kind of orders 1 and 3, respectively; and $\mu =2.07123\frac{\sin\varphi }{\sin(\varphi_{3\mathrm{dB}})}$, where $\varphi_{3\mathrm{dB}}$ and φ denote the 3 dB angle and the angle between R and the beam center relative to S, respectively.

The receive antenna gain for satellite parabolic antennas can be characterized by a piecewise approximation [52]

(4) $$\begin{array}{c} G_{b}\left(\mathrm{dB}\right)≃\left\{\begin{array}{cc} G_{\max}, & 0^{{}^{\circ}}<\varpi <1^{{}^{\circ}}\\ 32-25\log\varpi , & 1^{{}^{\circ}}<\varpi <48^{{}^{\circ}}\\ -10, & 48^{{}^{\circ}}<\varpi \leq 180^{{}^{\circ}} \end{array}\right., \end{array}$$

where $G_{\max}$ corresponds to the peak gain at boresight alignment, and ϖ indicates the angular deviation from the boresight direction.

The terrestrial relay R operates in a fixed-gain amplify-and-forward (AF) mode. Specifically, R amplifies the entire received electrical signal $y_{1}$, including both the desired signal and the FSO link noise $n_{1}$, by a fixed gain *G*, yielding the relay output $x_{R}=G\cdot y_{1}=G\left(\sqrt{P_{1}}\eta Hs+n_{1}\right)$. The amplified signal is then forwarded over the RF link. Consequently, the FSO noise $n_{1}$ propagates through the RF hop and contributes to the aggregate noise at the end receiver. In the subsequent RF link analysis and the end-to-end SNR derivation, this forwarded noise is properly accounted for through the relay gain parameter Υ, following the standard fixed-gain AF relaying framework [53].

In practice, CSI estimation is degraded by multipath effects, asymmetric scattering, and alignment mismatch between the directional FSO link and dynamic RF channel. These impairments are modeled using an additive CSI error framework [54]

(5) $$\begin{array}{c} \hat{H}=\rho H+\sqrt{1-\rho^{2}}\varepsilon , \end{array}$$

where ε∼CN(0,1) is independent of *H* and ρ∈(0,1] reflects the channel estimation accuracy (ρ=1: perfect CSI; ρ→0: uncorrelated estimate). The instantaneous SNR at R is $\gamma_{1}={\bar{\gamma }}_{1}{\left|\hat{H}\right|}^{t}$, where ${\bar{\gamma }}_{1}=\frac{\eta^{t}P_{1}}{\sigma_{1}^{2}}$ is the average SNR at R, and t=1 for HD or t=2 for IM/DD.

Assuming the channel coefficient of the FSO link follows the M distribution, the PDF and CDF of the instantaneous SNR $\gamma_{1}$ are obtained in a similar way as given in [37]

(6) $$\begin{array}{c} f_{\gamma_{1}}\left(\gamma \right)=\left\{\begin{array}{cc} \sum_{i=1}^{a}\sum_{k=0}^{\infty }A_{1}b_{i}\Delta \gamma^{\frac{k+1}{t}-1}e^{-\Omega \gamma^{\frac{2}{t}}}, & \gamma >0,\\ 1-K, & \gamma =0, \end{array}\right. \end{array}$$

(7) $$\begin{array}{c} F_{\gamma_{1}}\left(\gamma \right)=\left\{\begin{array}{cc} \sum_{i=1}^{a}\sum_{k=0}^{\infty }A_{1}A_{2}G_{1,2}^{1,1}\left[\Omega \gamma^{\frac{2}{t}}\left|\begin{array}{c} 1\\ \frac{1+k}{2},0 \end{array}\right.\right], & \gamma >0,\\ 1-K, & \gamma =0, \end{array}\right. \end{array}$$

where

(8) $$\begin{array}{cc} A_{1} & =\frac{2^{b-4.5}B\psi^{2}}{\pi^{1.5}}, \end{array}$$

(9) $$\begin{array}{cc} A_{2} & =\frac{2^{i+k-0.5}b_{i}G_{k}}{k!}, \end{array}$$

(10) $$\begin{array}{cc} Z & =\frac{ab}{\rho A_{0}h_{l}\left(\chi_{1}+ag\right)}, \end{array}$$

(11) $$\begin{array}{cc} \Omega & ={\left[2{\left({\bar{\gamma }}_{1}\right)}^{\frac{2}{t}}\left(1-\rho^{2}\right)\right]}^{-1}, \end{array}$$

(12) $$\begin{array}{cc} B & =\frac{2b^{0.5b}{\left(\frac{ag}{ag+\chi_{1}}\right)}^{0.5b+a}}{\Gamma \left(b\right)g^{1+0.5b}}, \end{array}$$

(13) $$\begin{array}{cc} \Delta & =\frac{G_{k}2^{i+0.5k}}{k!t{\left(1-\rho^{2}\right)}^{0.5(k+1)}{\bar{\gamma }}_{1}^{\frac{1+k}{t}}}, \end{array}$$

(14) $$\begin{array}{cc} K & =B\psi^{2}\sum_{i=1}^{a}\sum_{k=0}^{\infty }\frac{2^{k+i+b}G_{k}b_{i}\Gamma \left(\frac{1+k}{2}\right)}{32\pi^{1.5}k!}, \end{array}$$

(15) $$\begin{array}{cc} \chi_{1} & =\chi +2b_{0}\rho_{0}+2\sqrt{2b_{0}\rho_{0}\chi }\cos\left(\varphi_{A}-\varphi_{B}\right), \end{array}$$

(16) $$\begin{array}{cc} b_{i} & =\frac{\left(a-1\right)!a^{0.5i}\chi_{1}^{i-1}{\left(ag+\chi_{1}\right)}^{1-0.5i}}{g^{i-1}a^{0.5i}{\left[(i-1)!\right]}^{2}\left(a-i!\right)}\times {\left(\frac{ab}{ag+\chi_{1}}\right)}^{-0.5(b+i)}, \end{array}$$

and

(17) $$\begin{array}{cc} G_{k} & =G_{6,3}^{1,6}\left[\frac{8}{Z^{2}\left(1-\rho^{2}\right)}|\begin{array}{c} \frac{1-\psi^{2}}{2},\frac{2-\psi^{2}}{2},\frac{1-b}{2},\\ \frac{2-b}{2},\frac{1-i}{2},\frac{2-i}{2}\\ \frac{k}{2},-\frac{\psi^{2}}{2},\frac{1-\psi^{2}}{2} \end{array}\right]. \end{array}$$

*a* and *b* are atmospheric turbulence factors; $g=2b_{0}\left(1-\rho_{0}\right)$ is the average power of the independent scatter components that deviate from the transmission axis; $2b_{0}$ denotes the average power of the total scattering components, and $\rho_{0}$ is the ratio of scattering power coupled to the direct component; $\chi_{1}$ is the average power from the coherent contributions; χ is the average power of the direct component; $\varphi_{A}$ and $\varphi_{B}$ are the deterministic phase of the direct component and the scatter component coupled to the direct component, respectively.

From (7), we can obtain that as ${\bar{\gamma }}_{1}$ tends to infinity, the asymptotic CDF $F_{\gamma_{1}}^{\infty }\left(\gamma \right)≜\lim_{{\bar{\gamma }}_{1}\to \infty }F_{\gamma_{1}}\left(\gamma \right)$ satisfies $F_{\gamma_{1}}^{\infty }\left(\gamma_{th}\right)=1-K$, where $\gamma_{th}$ denotes a predetermined SNR threshold. This result is also equal to $F_{\gamma_{1}}\left(0\right)$ since K is independent of ${\bar{\gamma }}_{1}$ [37]. The basic reason is that the channel gain $\hat{H}$ must remain positive in practical systems, while estimation error ε may exhibit alternating polarity.

### 2.2. RF Link

The RF downlink employs omnidirectional transmission for broad coverage, which inherently exposes the signal to both U and potential eavesdroppers. Within the O-RAN architecture, the near-real-time (near-RT) RAN intelligent controller (RIC) hosts xApps/rApps that dynamically adjust RIS phase configurations based on real-time channel measurements. The RF link operates at 2.4 GHz in the ISM band, compatible with low-cost RIS implementations using typical varactor-based designs. Under imperfect CSI, the received signals at U and E are modeled as

(18) $$\begin{array}{c} y_{x}=\sum_{n=1}^{N}\sqrt{P_{2}}\eta \hat{H}{\hat{g}}_{n}e^{j\theta_{n}}{\hat{h}}_{n}^{x}s+n_{x}, \end{array}$$

where $P_{2}$ is the transmit power of R; $x\in \left\{u,e\right\}$; n∈{1,2,…,N}; *N* is the number of the reflective elements of RIS; $\theta_{n}$ is the *n*-th reflective element of the RIS; $n_{x}∼\mathrm{CN}\left(0,\sigma_{x}^{2}\right)$ is the AWGN at the U/E; ${\hat{g}}_{n}$ and ${\hat{h}}_{n}^{x}$ are the imperfect channel gains of the *n*-th element for R-RIS and RIS-U/E link, respectively. Note that $\hat{H}$ in (18) refers to the same estimated FSO channel gain defined in (5). This coupling arises because the AF relay amplifies and forwards the received optical signal, thereby embedding the FSO channel estimate into the subsequent RF transmission.

In RIS-assisted satellite communication systems, acquiring accurate CSI is fundamentally challenging because the RIS elements are passive devices that cannot transmit, receive, or process pilot signals independently. The channel coefficients for the R-RIS and RIS-U/E links are typically estimated as cascaded channels through a two-stage pilot-based protocol. In the first stage, the RIS elements are sequentially configured to known phase states while the relay transmits pilot symbols, and the user measures the received signals to estimate the cascaded channel gains corresponding to each RIS element. In the second stage, the RIS phase shifts are optimized using the estimated cascaded CSI, and the effective end-to-end channel is refined. For satellite downlink scenarios, the long propagation delay, the high Doppler shift induced by satellite motion, and the limited feedback bandwidth from terrestrial terminals to the satellite further degrade the CSI estimation accuracy, making imperfect CSI a practically inevitable condition rather than a mere theoretical assumption. The adopted imperfect CSI model is given by

(19) $$\begin{array}{c} {\hat{g}}_{n}=\rho g_{n}+\sqrt{1-\rho^{2}}\varepsilon , \end{array}$$

(20) $$\begin{array}{c} {\hat{h}}_{n}^{x}=\rho h_{n}^{x}+\sqrt{1-\rho^{2}}\varepsilon , \end{array}$$

where $g_{n}=L_{2}\left(d_{r}\right)\alpha_{n}e^{-j\zeta_{n}}$ and $h_{n}^{x}=L_{2}\left(d_{x}\right)\beta_{n}^{x}e^{-j\xi_{n}^{x}}$ are the actual channel gains of the *n*-th element for R-RIS and RIS-U/E link, respectively, with $\alpha_{n}$, $\beta_{n}^{x}$ being the channel amplitudes following the Rayleigh distribution, $\zeta_{n}$, $\xi_{n}$ the channel phases and $L_{2}\left(d\right)=C_{0}{\left(\frac{d}{D_{0}}\right)}^{\tau }$ the path loss. Here $C_{0}$ is the path loss at the reference distance $D_{0}=1$ kilometer (km); $d_{r}$ and $d_{x}$ are the distance of R-RIS and RIS-U/E link, respectively; τ denotes the path loss factor. Furthermore, since the eavesdropper is passive and does not cooperate with the legitimate network, its CSI is typically even more difficult to estimate accurately. We note that the model adopts a single channel estimation accuracy coefficient ρ and a common error variable ε∼CN(0,1) across the FSO link (5) and all RF sub-links (19) and (20). Although this unified treatment preserves analytical tractability while capturing the essential impact of imperfect CSI on the end-to-end secrecy performance, it entails two simplifying assumptions that warrant discussion. First, the FSO and RF hops operate over fundamentally different physical media (optical versus radio), employ distinct channel estimation protocols, and experience independent error realizations; a more refined model would assign hop-specific accuracy coefficients (e.g., $\rho_{\mathrm{FSO}}$, $\rho_{\mathrm{RF}}$) and independent error variables ($\varepsilon_{\mathrm{FSO}}$, $\varepsilon_{\mathrm{RF}}$). Second, the model assumes identical CSI quality for the legitimate user and the eavesdropper. Since the eavesdropper is passive and non-cooperative, its CSI is typically worse than that of the legitimate user; assuming equal ρ therefore represents a conservative worst-case benchmark from the security perspective. By treating ρ as a tunable system parameter, our analysis enables a systematic investigation of how CSI quality affects the secrecy performance and provides practical guidelines for determining the required channel estimation accuracy to meet specific security requirements. Extending the framework to incorporate heterogeneous ρ values and independent error variables across links represents an important direction for future investigation, at the cost of increased notational and analytical complexity.

In the considered satellite downlink scenario, the terrestrial terminals (U and E) are typically situated in complex urban or suburban environments characterized by dense local scatterers such as buildings, vehicles, and foliage. Although the RIS may be deployed at a strategically favorable location (e.g., on a tall building facade) to establish a LoS path toward the relay R, the RIS-to-U and RIS-to-E links are ground-level channels where the LoS component can be substantially attenuated or completely obstructed by the surrounding clutter. Consequently, Rayleigh fading serves as a physically reasonable and analytically tractable model for characterizing the composite small-scale fading along the RIS-assisted RF links under rich-scattering conditions. We note that in scenarios where a strong LoS component is expected from the RIS to the terminals (e.g., open rural areas or dedicated aerial receivers), the Rician fading model would provide a more accurate statistical characterization. Extending the proposed analytical framework to incorporate Rician fading for the RIS-assisted RF links constitutes an important direction for future investigation, which would enable the quantification of the security enhancement afforded by a dominant specular path.

Substituting the optimal phases into the composite channel expression and accounting for imperfect CSI, we derive the instantaneous SNR at U/E through algebraic manipulation and leveraging the statistical properties of the channels, as given by

(21) $$\begin{array}{cc} \gamma_{2}^{x} & ={\left|\sum_{n=1}^{N}\rho^{3}\alpha_{n}\beta_{n}^{x}\eta HL_{2}\left(d_{r}\right)L_{2}\left(d_{x}\right)\right|}^{2}\;\times [H_{\rho }(\rho^{2}\left(1-\rho^{2}\right)|\sum_{n=1}^{N}\alpha_{n}e^{j\xi_{n}^{x}}L_{2}\left(d_{r}\right){|}^{2}\\ \; & \;+\rho^{2}\left(1-\rho^{2}\right)|\sum_{n=1}^{N}\beta_{n}^{x}e^{j\zeta_{n}}L_{2}\left(d_{x}\right){|}^{2}\;+N{\left(1-\rho^{2}\right)}^{2}|L_{2}\left(d_{r}\right)L_{2}\left(d_{x}\right){|}^{2})+\frac{\sigma_{x}^{2}}{P_{2}}{]}^{-1}. \end{array}$$

where $H_{\rho }=\eta^{2}\left(1-\rho^{2}+\rho^{2}H^{2}\right)$. Furthermore, we define the average SNR at U/E as ${\bar{\gamma }}_{2}^{x}=E\left[\gamma_{2}^{x}\right]$.

To address random link blockage caused by electromagnetic interference and physical obstructions [55], we model the RF channel with reflective path blocking probability $p_{0}$. This binary blockage model provides a tractable framework for initial secrecy performance analysis, which is applied exclusively to the RIS-assisted reflective links (RIS-U and RIS-E). The direct links are treated as permanently blocked and are not assigned a probabilistic blockage model. Leveraging small-argument approximations, the numerator in (21) follows a squared $K_{G}$ distribution while the denominator obeys Gamma distribution. This approach establishes the statistical models that enable derivation of the PDF and CDF for $\gamma_{2}^{x}$ under link blockage conditions, as follows:

(22) $$f_{\gamma_{2}^{x}}\left(\gamma \right)=p_{0}\delta \left(\gamma \right)+\frac{\left(1-p_{0}\right)G_{1,2}^{2,1}\left[\frac{\Lambda^{2}\gamma }{\rho^{2}u_{x}}|\begin{array}{c} 1-\nu_{x}\\ m_{1},m_{2} \end{array}\right]}{\Gamma \left(m_{1}\right)\Gamma \left(m_{2}\right)\Gamma \left(\nu_{x}\right)\gamma },$$

(23) $$F_{\gamma_{2}^{x}}\left(\gamma \right)=p_{0}+\frac{\left(1-p_{0}\right)G_{2,3}^{2,2}\left[\frac{\Lambda^{2}\gamma }{\rho^{2}u_{x}}|\begin{array}{c} 1-\nu_{x},1\\ m_{1},m_{2},0 \end{array}\right]}{\Gamma \left(m_{1}\right)\Gamma \left(m_{2}\right)\Gamma \left(\nu_{x}\right)},$$

where

(24) $$m_{1}=\frac{-b_{w}+\sqrt{b_{w}^{2}-4a_{w}c_{w}}}{2a_{w}},$$

(25) $$m_{2}=\frac{-b_{w}-\sqrt{b_{w}^{2}-4a_{w}c_{w}}}{2a_{w}},$$

$m_{1}$ and $m_{2}$ are the shaping parameters; $\Lambda =\sqrt{m_{1}m_{2}/\Omega_{w}}$; $\Omega_{w}$, $a_{w}$, $b_{w}$ and $c_{w}$ have been defined in [56]; $u_{x}$ and $\nu_{x}$ are expressed as

(26) $$u_{x}=\frac{1}{\Xi_{x}}\left[1+\frac{1}{2\Xi_{x}}\left(\Xi_{x}+\frac{\sigma_{x}^{2}}{P_{2}G_{2}^{x}}\right)\right],$$

(27) $$\nu_{x}=2\left[1+\frac{1}{2\Xi_{x}}\left(\Xi_{x}+\frac{\sigma_{x}^{2}}{P_{2}G_{2}^{x}}\right)\right],$$

where

(28) $$\begin{array}{c} \Xi_{x}=H_{\rho }N{\left(1-\rho^{2}\right)}^{2}+P_{2}G_{2}^{x}|L_{2}\left(d_{r}\right)L_{2}\left(d_{x}\right){|}^{2}\sigma_{x}^{-2}. \end{array}$$

The binary blockage model with probability $p_{0}$ is adopted as a first-order approximation to maintain analytical tractability while capturing the essential impact of blockages on system performance. In this work, we assume that the blockage events on the legitimate user’s link (RIS-U) and the eavesdropper’s link (RIS-E) are statistically independent and identically distributed with the same blocking probability $p_{0}$. This independence assumption is physically justified when U and E are located at sufficiently separated positions, such that the obstacles intermittently blocking one link are uncorrelated with those affecting the other. Under this model, the joint blockage probability for both links is $p_{0}^{2}$, while the probability that at least one link is unblocked is $1-p_{0}^{2}$. The independence structure is implicitly used in the derivation of (22) and (23), where the blockage indicator for each receiver is applied separately. For scenarios where blockages are spatially correlated (e.g., U and E are in close proximity behind a common obstacle), a more general correlated blockage model would be required, which constitutes an interesting extension for future work.

## 3. Reliability and Secrecy Performance Analysis

This section derives the closed-form expressions for the COP as a reliability metric, along with SOP and PPSC as the primary secrecy performance metrics. These analytical expressions can serve as the theoretical foundation for security-oriented xApps in the O-RAN near-RT RIC, enabling real-time secrecy assessment. To further uncover fundamental performance limits, high-SNR asymptotic analyses are conducted, characterizing the intrinsic security boundaries of the channel. For secrecy performance analysis, we adopt the fixed-gain amplify-and-forward (AF) relaying strategy, which can achieve the diversity and multiplexing trade-off upper bound [53].

### 3.1. COP Analysis

The COP characterizes the service connectivity of the satellite downlink transmission system, quantifying the probability that the end-to-end instantaneous SNR $\gamma_{3}=\frac{\gamma_{1}\gamma_{2}^{u}}{\gamma_{2}^{u}+\Upsilon }$ at the intended receiver falls below a predetermined threshold $\gamma_{th}$ [57], thereby failing to establish a reliable communication link. As a reliability metric rather than a secrecy metric, COP measures the system’s ability to maintain basic communication quality, and its closed-form expression serves as the foundation for the subsequent secrecy performance analysis. Consequently, the COP for the fixed-gain AF relay at U is expressed as

(29) $$\begin{array}{c} P_{cop}\left(\gamma_{th}\right)=P\left(\frac{\gamma_{1}\gamma_{2}^{u}}{\gamma_{2}^{u}+\Upsilon }<\gamma_{th}\right), \end{array}$$

where Υ is a constant determined by the relay’s fixed gain *G* and the FSO link noise statistics. This parameter encapsulates the contribution of the forwarded FSO noise $n_{1}$ to the end-to-end SNR, which is a defining characteristic of the fixed-gain AF relaying protocol. To streamline the analysis, we apply the variable substitution $\omega =\gamma_{1}-\gamma_{th}$, decoupling the SNR components. Consequently, (29) can be reformulated as

(30) $$\begin{array}{c} P_{cop}\left(\gamma_{th}\right)=F_{\gamma_{1}}\left(\gamma_{th}\right)+\int_{0}^{\infty }F_{\gamma_{2}^{u}}\left(\frac{\Upsilon \gamma_{th}}{\omega }\right)f_{\gamma_{1}}\left(\gamma_{th}+\omega \right)d\omega . \end{array}$$

Using the binary Fox-*H* function $H_{p,q}^{m,n}\left[\cdot \right]$ defined in [58] (Equations (1.2) and (1.3)) and substituting (6), (7) and (23) into (30), we derive the closed-form expression for $P_{cop}\left(\gamma_{th}\right)$ as shown in (31).

(31) $$\begin{array}{cc} P_{cop}\left(\gamma_{th}\right) & =p_{0}+\left(1-p_{0}\right)\left(\sum_{i=1}^{a}\sum_{k=0}^{\infty }A_{1}A_{2}G_{1,2}^{1,1}\left[\Omega \gamma_{th}^{\frac{2}{t}}\left|\begin{array}{c} 1\\ \frac{1+k}{2},0 \end{array}\right.\right]\right)\\ \; & \;+\left(1-p_{0}\right)\sum_{i=1}^{a}\sum_{k=0}^{\infty }\frac{A_{1}b_{i}\Delta }{\Gamma \left(m_{1}\right)\Gamma \left(m_{2}\right)\Gamma \left(\nu_{u}\right)}\\ \; & \;\times H_{1,0;1,3;2,2}^{0,1;2,2;1,1}[\begin{array}{c} \left(1;\frac{2}{t},-1\right)\\ - \end{array}|\begin{array}{c} \left(1,1\right)\\ \left(\frac{1+k}{2},1\right),\left(1,\frac{2}{t}\right),\left(0,1\right) \end{array}|\\ \; & \;\begin{array}{c} \left(1-\nu_{u},1\right),\left(1,1\right)\\ \left(m_{1},1\right),\left(m_{2},1\right) \end{array}|\begin{array}{c} \Omega \gamma_{th}^{\frac{2}{t}}\\ \frac{\Lambda^{2}\Upsilon \gamma_{th}}{\rho^{2}u_{u}} \end{array}]. \end{array}$$

**Proof.**  The detailed proof is provided in Appendix A and the derivation proceeds through three key steps:

(1) Transforming the product of exponential and power functions in (30) into Mellin–Barnes contour integrals.
(2) Applying the Meijer-*G* function representation [59] (Equation (9.301)) to decompose complex factors.
(3) Evaluating the resulting contour integral using residue calculus and convergence analysis via Mellin–Barnes techniques [60] (Equation (1.1)).

This completes the proof. □

**Remark** **1.** *From* (31)*, when the link blockage probability $p_{0}=1$ (i.e., the RF link is fully blocked), $P_{cop}=1$, indicating that U cannot establish a reliable communication link to R under full RF link blockage. This extreme condition reveals a fundamental limitation of the satellite downlink transmission system: complete signal discontinuity occurs when environmental blockages sever all RIS-assisted propagation paths to the authorized receiver.*

We now analyze the asymptotic behavior of COP under high-SNR regimes. As the average SNRs ${\bar{\gamma }}_{1}$ and ${\bar{\gamma }}_{2}^{u}\to \infty$, the asymptotic COP approximation simplifies to

(32) $$\begin{array}{c} P_{cop}^{\infty }=F_{\gamma_{1}}^{\infty }\left(\gamma_{th}\right)+\int_{0}^{\infty }F_{\gamma_{2}^{u}}^{\infty }\left(\frac{\Upsilon \gamma_{th}}{\omega }\right)f_{\gamma_{1}}^{\infty }\left(\gamma_{th}+\omega \right)d\omega . \end{array}$$

Leveraging asymptotic expansions of Fox-*H* functions [61] (Equations (1.5.9) and (1.8.4)) and applying [60] (Equation (1.1)), the asymptotic COP expression is derived as shown in (33).

(33) $$\begin{array}{cc} \; & P_{cop}^{\infty }\left(\gamma_{th}\right)=p_{0}+\left(1-p_{0}\right)\left[1-B\psi^{2}\sum_{i=1}^{a}\sum_{k=0}^{\infty }\frac{2^{k+i+b}G_{k}b_{i}\Gamma \left(\frac{1+k}{2}\right)}{32\pi^{1.5}k!}\right]\\ \; & \;+\left(1-p_{0}\right)\sum_{i=1}^{a}\sum_{k=0}^{\infty }\frac{A_{1}b_{i}\Delta }{\Gamma \left(m_{1}\right)\Gamma \left(m_{2}\right)\Gamma \left(\nu_{u}\right)}\times \\ \; & (\Gamma \left(\frac{1+k}{2}-\frac{t}{2}\right){\left(\Omega \gamma_{th}^{\frac{2}{t}}\right)}^{\frac{t}{2}}G_{1,2}^{2,1}\left[\frac{\Lambda^{2}\Upsilon \gamma_{th}}{\rho^{2}u_{u}}|\begin{array}{c} 1-\nu_{u}\\ m_{1},m_{2} \end{array}\right]\\ \; & \;+\frac{2\Gamma \left(1-\frac{1+k}{t}\right)}{1+k}{\left(\Omega \gamma_{th}^{\frac{2}{t}}\right)}^{\frac{1+k}{2}}G_{2,3}^{3,1}\left[\frac{\Lambda^{2}\Upsilon \gamma_{th}}{\rho^{2}u_{u}}|\begin{array}{c} 1-\nu_{u},1\\ m_{1},m_{2},\frac{1+k}{t} \end{array}\right]). \end{array}$$

**Proof.**  The detailed proof is provided in Appendix B. To derive the asymptotic COP under high-SNR conditions, we rigorously apply Mellin–Barnes contour integration and Meijer-*G* function approximations. The derivation proceeds through three key steps:

(1) Expressing the exact COP solution from (31) in its Mellin–Barnes integral representation using the Fox-*H* function definition [61] (Equation (1.1.2)).
(2) Deforming the integration contour in the complex plane to isolate dominant poles governing high-SNR behavior.
(3) Applying the asymptotic expansion formulas for Fox-*H* functions [61] (Equations (1.5.9) and (1.8.4)), which leverage the residue theorem at these critical poles, we extract the leading-order terms.

This completes the proof. □

Through algebraic simplification of the residue series, we consolidate the asymptotic expression. This yields the closed-form expression for $P_{\mathrm{cop}}^{\infty }\left(\gamma_{\mathrm{th}}\right)$ shown in (33).

**Remark** **2.** *From* (7)*, $F_{\gamma_{1}}^{\infty }\left(\gamma_{th}\right)=1-K$ [37]. Furthermore, $F_{\gamma_{2}^{u}}^{\infty }\left(\frac{\Upsilon \gamma_{th}}{\omega }\right)=F_{\gamma_{2}^{u}}^{\infty }\left(0\right)$, which remains constant. Given the integral property $\int_{0}^{\infty }f_{\gamma_{1}}\left(\gamma \right)d\gamma =1$, it follows that $P_{cop}$ converges to a constant as ${\bar{\gamma }}_{1}$ and ${\bar{\gamma }}_{2}^{u}\to \infty$.*

### 3.2. SOP Analysis

The SOP quantifies the probability of confidentiality failure, which is defined as the event where the instantaneous secrecy capacity (SC) of the RF channel falls below the predefined secure threshold rate $R_{th}$. Here, the secrecy capacity $C_{s}≜\max\left\{0,\log_{2}\left(1+\gamma_{2}^{u}\right)-\log_{2}\left(1+\gamma_{2}^{e}\right)\right\}$ represents the maximum achievable secure rate under given channel realizations, while $R_{th}>0$ is a fixed threshold selected by the system designer. It thus functions as a vital metric for assessing and designing secure communication systems. For a fixed-gain AF relay, the SOP of the considered system is expressed as

(34) $$\begin{array}{cc} P_{sop} & =P\left({\left[\log_{2}\left(1+\gamma_{2}^{u}\right)-\log_{2}\left(1+\gamma_{2}^{e}\right)\right]}^{+}<R_{th}\right)\\ \; & =\int_{0}^{\infty }P_{cop}\left(\gamma \cdot \varrho \right)f_{\gamma_{2}^{e}}\left(\gamma \right)d\gamma , \end{array}$$

where ${\left[x\right]}^{+}=\max\left(x,0\right)$ and $\varrho =2^{R_{th}}$.

Substituting (7), (22) and (31) into (34), we similarly derive the closed-form expression for $P_{sop}$ as shown in (35), where the parameter $K_{p_{0}}$ is defined as $K_{p_{0}}=p_{0}+K\left(1-p_{0}\right)p_{0}$.

(35) $$\begin{array}{cc} P_{sop}= & K_{p_{0}}+\sum_{i=1}^{a}\sum_{k=0}^{\infty }\frac{{\left(1-p_{0}\right)}^{2}A_{1}A_{2}}{\Gamma \left(m_{1}\right)\Gamma \left(m_{2}\right)\Gamma \left(\nu_{e}\right)}H_{2,3}^{2,2}\left[\begin{array}{c} \Omega \varrho^{\frac{2}{t}}\\ \frac{\Lambda^{2}}{\rho^{2}u_{e}} \end{array}|\begin{array}{c} \left(1-\nu_{e},1\right),\left(1,1\right)\\ \left(m_{1},1\right),\left(m_{2},1\right),\left(0,1\right) \end{array}\right]\\ \; & +\sum_{i=1}^{a}\sum_{k=0}^{\infty }\frac{A_{1}b_{i}\Delta {\left[\Gamma \left(\nu_{u}\right)\Gamma \left(\nu_{e}\right)\right]}^{-1}}{\Gamma {\left(m_{1}\right)}^{2}\Gamma {\left(m_{2}\right)}^{2}{\left(1-p_{0}\right)}^{-2}}\times H_{1,0;1,3;3,2}^{0,1;2,1;2,2}[\begin{array}{c} \left(1;\frac{2}{t},-1\right)\\ - \end{array}|\begin{array}{c} \left(1,1\right)\\ \left(\frac{1+k}{2},1\right),\left(1,\frac{2}{t}\right),\left(0,1\right) \end{array}|\\ \; & \begin{array}{c} \left(1-\nu_{u},1\right),\left(1-\nu_{e},1\right),\left(1,1\right)\\ \left(m_{1},1\right),\left(m_{2},1\right) \end{array}|\begin{array}{c} \Omega \varrho \Upsilon^{\frac{2}{t}}\\ \frac{\Lambda^{2}\varrho }{\rho^{2}u_{u}u_{e}} \end{array}]. \end{array}$$

**Proof.**  See Appendix C. The derivation proceeds through three key steps:

(1) Decomposing the SOP integral into delta function and continuous components, with the delta component evaluated using the sifting property.
(2) Transforming integral components using Meijer-*G* function product formulas and Mellin transforms to obtain the bivariate Fox-*H* function and the extended generalized bivariate *H* function.
(3) Combining all analytical results through rigorous algebraic manipulation to construct the final closed-form expression in (35).

This completes the proof. □

**Remark** **3.** *When the blocking probability of RF channel attains $p_{0}=1$ (i.e., complete obstruction of the omnidirectional transmission path), $P_{sop}=1$ manifests as all authorized receivers suffer service dropout. This total RF link blockage annihilates secure communication capability, collapsing secrecy capacity to zero, highlighting the inherent vulnerability of communication systems to environmental blockages, where the open-air propagation advantage paradoxically becomes a security liability. It is important to note that the derived SOP expression corresponds to a lower bound on the exact secrecy outage probability. This bound is obtained through the widely adopted approximation $\frac{1+\gamma_{2}^{u}}{1+\gamma_{2}^{e}}\approx \frac{\gamma_{2}^{u}}{\gamma_{2}^{e}}$, which is tight in the medium-to-high-SNR regime of practical interest. Consequently, the actual secrecy outage performance is guaranteed to be no worse than the analytical results presented in this work, and the derived expressions provide a conservative yet accurate characterization of the system’s security performance.*

As ${\bar{\gamma }}_{1}$ and ${\bar{\gamma }}_{2}^{u}\to \infty$, the asymptotic SOP is expressed as

(36) $$\begin{array}{c} P_{sop}^{\infty }=\int_{0}^{\infty }P_{cop}^{\infty }\left(\gamma \cdot \varrho \right)f_{\gamma_{2}^{e}}\left(\gamma \right)d\gamma , \end{array}$$

where $P_{cop}^{\infty }$ is constant. From (35), the first two terms are independent of ${\bar{\gamma }}_{2}^{u}$. Therefore, based on the asymptotic expansion presented in [61] (Equation (1.8.4)), the asymptotic SOP expression can be similarly derived as given in (37).

(37) $$\begin{array}{cc} \; & P_{sop}^{\infty }=K_{p_{0}}+\sum_{i=1}^{a}\sum_{k=0}^{\infty }\frac{{\left(1-p_{0}\right)}^{2}A_{1}A_{2}}{\Gamma \left(m_{1}\right)\Gamma \left(m_{2}\right)\Gamma \left(\nu_{e}\right)}H_{2,3}^{2,2}\left[\begin{array}{c} \Omega \varrho^{\frac{2}{t}}\\ \frac{\Lambda^{2}}{\rho^{2}u_{e}} \end{array}|\begin{array}{c} \left(1-\nu_{e},1\right),\left(1,1\right)\\ \left(m_{1},1\right),\left(m_{2},1\right),\left(0,1\right) \end{array}\right]\\ \; & +\sum_{i=1}^{a}\sum_{k=0}^{\infty }\frac{A_{1}b_{i}\Delta {\left[\Gamma \left(\nu_{u}\right)\Gamma \left(\nu_{e}\right)\right]}^{-1}}{\Gamma {\left(m_{1}\right)}^{2}\Gamma {\left(m_{2}\right)}^{2}{\left(1-p_{0}\right)}^{-2}}\times (\frac{2\Gamma \left(1-\frac{1+k}{t}\right)}{\left(1+k\right){\left(\Omega \varrho \right)}^{-\frac{1+k}{2}}\Upsilon^{-\frac{1+k}{t}}}G_{2,4}^{3,2}\left[\frac{\Lambda^{2}\varrho }{\rho^{2}u_{u}u_{e}}|\begin{array}{c} 1-\nu_{u},1-\nu_{e}\\ m_{1},m_{2},\frac{1+k}{t},1 \end{array}\right]\;\\ \; & +\Gamma \left(\frac{1+k}{2}-\frac{t}{2}\right){\left(\Omega \varrho \right)}^{\frac{t}{2}}\Upsilon G_{2,2}^{2,2}\left[\frac{\Lambda^{2}\varrho }{\rho^{2}u_{u}u_{e}}|\begin{array}{c} 1-\nu_{u},1-\nu_{e}\\ m_{1},m_{2} \end{array}\right]). \end{array}$$

**Proof.**  See Appendix D. The derivation proceeds through three key steps:

(1) Establishing the integral representation by converting the extended generalized bivariate *H* function into its Mellin–Barnes double integral form using [59] (Equation (3.381.4)).
(2) Identifying the dominant poles by analyzing the Gamma functions in the integrand to determine the poles with smallest real parts that dominate asymptotic behavior.
(3) Applying the residue theorem to evaluate the dominant pole contributions following [60] (Equation (1.1)) and [61] (Equation (1.8.4)), thereby reducing the complex bivariate function to simpler Meijer-*G* function terms as in (37).

This completes the proof. □

To gain deeper insights into the high-SNR scaling behavior, we investigate the corresponding secrecy diversity order. The secrecy diversity order is defined as

(38) $$\begin{array}{c} D_{s}=-\lim_{{\bar{\gamma }}_{2}^{u}\to \infty }\frac{\log P_{sop}}{\log{\bar{\gamma }}_{2}^{u}}, \end{array}$$

which quantifies how rapidly the SOP decays as the average SNR of the RF link increases. Substituting (33) into (36) and noting that $\int_{0}^{\infty }f_{\gamma_{2}^{e}}\left(\gamma \right)d\gamma =1$, we find that $P_{sop}$ converges to a non-zero constant floor given by

(39) $$\begin{array}{c} F_{sop}\left(p_{0},\rho \right)=p_{0}+\left(1-p_{0}\right)\left[1-K(\rho )\right]. \end{array}$$

where K(ρ) is the CSI error floor defined in (14). Consequently, the secrecy diversity order is given by $D_{s}=0$.

**Remark** **4.** *This result indicates that in the high-SNR regime and under practical deployment constraints, the secrecy performance floor is jointly dominated by link blockage and imperfect CSI, both of which are SNR-independent. Consequently, the secrecy diversity order is zero, and increasing the transmit power yields diminishing returns once the RIS beamforming gain is sufficient. In the finite-SNR regime before saturation, the RIS array gain and CSI accuracy jointly dictate the coding gain, determining how quickly the floor is approached.*

In the limiting case of perfect CSI (ρ=1), we have $F_{sop}\left(p_{0},1\right)=p_{0}+\left(1-p_{0}\right)\left[1-K(1)\right]$, where the floor is dominated by $p_{0}$ and the residual term is determined by the FSO link outage floor K(1). For imperfect CSI (ρ<1), the floor is elevated by the additional term proportional to $\left(1-p_{0}\right)$, which quantifies the secrecy penalty due to channel estimation errors. This stands in sharp contrast to the results in [30,31,35], where perfect CSI was assumed in the absence of link blockage, leading to non-zero diversity orders. The zero diversity order found here provides a practically important caution: in realistic deployments, mitigating link blockage and ensuring sufficient CSI accuracy are essential, as these impairments ultimately dictate the achievable secrecy performance.

### 3.3. PPSC Analysis

Based on Wyner’s wiretap framework adapted for communication systems [35], the PPSC is a key security metric that evaluates the probability of achieving secure transmission with a non-zero secrecy rate, i.e., $P(C_{s}>0)$, which represents the fundamental precondition for secure communication. In contrast, SOP imposes a stricter requirement by testing whether $C_{s}$ exceeds a positive target rate $R_{th}$. In the limiting case $R_{th}\to 0$, we have $P_{sop}\to 1-P_{ppsc}$, indicating that PPSC corresponds to the best-case secrecy outage performance. For $R_{th}>0$, $P_{sop}\geq 1-P_{ppsc}$, with the gap quantifying the degradation caused by the non-zero rate requirement. These two metrics thus provide complementary perspectives: PPSC reflects whether the legitimate channel dominates the eavesdropper’s channel, while SOP measures the reliability of supporting a desired secure rate. The PPSC is formally defined as follows:

(40) $$\begin{array}{cc} P_{ppsc} & =P\left({\left[\log_{2}\left(1+\gamma_{2}^{u}\right)-\log_{2}\left(1+\gamma_{2}^{e}\right)\right]}^{+}>0\right)\\ \; & =1-\int_{0}^{\infty }P_{cop}\left(\gamma \right)f_{\gamma_{2}^{e}}\left(\gamma \right)d\gamma \\ \; & =1-P_{sop}{|}_{\varrho =1}. \end{array}$$

Substituting (35) into (40), we can derive the closed-form expression for $P_{ppsc}$ as shown in (41).

(41) $$\begin{array}{cc} P_{ppsc}= & 1-K_{p_{0}}-\sum_{i=1}^{a}\sum_{k=0}^{\infty }\frac{{\left(1-p_{0}\right)}^{2}A_{1}A_{2}}{\Gamma \left(m_{1}\right)\Gamma \left(m_{2}\right)\Gamma \left(\nu_{e}\right)}H_{2,3}^{2,2}\left[\begin{array}{c} \Omega \\ \frac{\Lambda^{2}}{\rho^{2}u_{e}} \end{array}|\begin{array}{c} \left(1-\nu_{e},1\right),\left(1,1\right)\\ \left(m_{1},1\right),\left(m_{2},1\right),\left(0,1\right) \end{array}\right]\\ \; & -\sum_{i=1}^{a}\sum_{k=0}^{\infty }\frac{A_{1}b_{i}\Delta {\left[\Gamma \left(\nu_{u}\right)\Gamma \left(\nu_{e}\right)\right]}^{-1}}{\Gamma {\left(m_{1}\right)}^{2}\Gamma {\left(m_{2}\right)}^{2}{\left(1-p_{0}\right)}^{-2}}\times H_{1,0;1,3;3,2}^{0,1;2,1;2,2}[\begin{array}{c} \left(1;\frac{2}{t},-1\right)\\ - \end{array}|\begin{array}{c} \left(1,1\right)\\ \left(\frac{1+k}{2},1\right),\left(1,\frac{2}{t}\right),\left(0,1\right) \end{array}|\\ \; & \begin{array}{c} \left(1-\nu_{u},1\right),\left(1-\nu_{e},1\right),\left(1,1\right)\\ \left(m_{1},1\right),\left(m_{2},1\right) \end{array}|\begin{array}{c} \Omega \Upsilon^{\frac{2}{t}}\\ \frac{\Lambda^{2}}{\rho^{2}u_{u}u_{e}} \end{array}]. \end{array}$$

As ${\bar{\gamma }}_{1}$ and ${\bar{\gamma }}_{2}^{u}\to \infty$, PPSC converges to a constant close to 1 and $P_{ppsc}^{\infty }=1-P_{sop}^{\infty }{|}_{\varrho =1}$. Using (35) and (37), we similarly derive the asymptotic expressions for $P_{ppsc}$ as shown in (42).

(42) $$\begin{array}{cc} \; & P_{ppsc}^{\infty }=1-K_{p_{0}}-\sum_{i=1}^{a}\sum_{k=0}^{\infty }\frac{{\left(1-p_{0}\right)}^{2}A_{1}A_{2}}{\Gamma \left(m_{1}\right)\Gamma \left(m_{2}\right)\Gamma \left(\nu_{e}\right)}H_{2,3}^{2,2}\left[\begin{array}{c} \Omega \\ \frac{\Lambda^{2}}{\rho^{2}u_{e}} \end{array}|\begin{array}{c} \left(1-\nu_{e},1\right),\left(1,1\right)\\ \left(m_{1},1\right),\left(m_{2},1\right),\left(0,1\right) \end{array}\right]\\ \; & -\sum_{i=1}^{a}\sum_{k=0}^{\infty }\frac{A_{1}b_{i}\Delta {\left[\Gamma \left(\nu_{u}\right)\Gamma \left(\nu_{e}\right)\right]}^{-1}}{\Gamma {\left(m_{1}\right)}^{2}\Gamma {\left(m_{2}\right)}^{2}{\left(1-p_{0}\right)}^{-2}}\times (\frac{2\Gamma \left(1-\frac{1+k}{t}\right)}{\left(1+k\right){\left(\Omega \right)}^{-\frac{1+k}{2}}\Upsilon^{-\frac{1+k}{t}}}G_{2,4}^{3,2}\left[\frac{\Lambda^{2}}{\rho^{2}u_{u}u_{e}}|\begin{array}{c} 1-\nu_{u},1-\nu_{e}\\ m_{1},m_{2},\frac{1+k}{t},1 \end{array}\right]\\ \; & \;+\Gamma \left(\frac{1+k}{2}-\frac{t}{2}\right){\left(\Omega \right)}^{\frac{t}{2}}\Upsilon G_{2,2}^{2,2}\left[\frac{\Lambda^{2}}{\rho^{2}u_{u}u_{e}}|\begin{array}{c} 1-\nu_{u},1-\nu_{e}\\ m_{1},m_{2} \end{array}\right]). \end{array}$$

### 3.4. Discrete Phase Shift Analysis

The analysis presented thus far assumes ideal continuous RIS phase shift control. However, practical RIS elements rely on discrete-component phase shifters (e.g., PIN diodes or varactors) that can only realize a finite set of quantized phase states due to fabrication cost, circuit complexity, and power consumption constraints. To bridge the gap between theoretical analysis and hardware reality, this subsection quantifies the secrecy performance loss incurred by discrete phase constraints. In practical deployments, the RIS is limited to discrete phase adjustments with $2^{q}$ quantization levels, where *q* denotes the number of quantization bits per reflecting element. This process is typically modeled as a uniform quantization of the continuous phase range [0,2π). The RIS phase mismatch is defined as the difference between the ideal continuous phase and the quantized phase, i.e., $\Delta \theta =\theta_{\mathrm{ideal}}-\theta_{\mathrm{quantized}}$. To quantify the impact of discrete phase shifts, we model the phase quantization error Δθ as a uniformly distributed random variable over the interval $\left[-\pi /2^{q},\pi /2^{q}\right]$. Its PDF is given by:

(43) $$\begin{array}{c} f_{\Delta \theta }\left(\theta \right)=\left\{\begin{array}{cc} \frac{2^{q}}{2\pi }, & -\frac{\pi }{2^{q}}\leq \theta \leq \frac{\pi }{2^{q}},\\ 0, & \mathrm{otherwise}. \end{array}\right.. \end{array}$$

Based on the approximation Equation (18) in [62], the combined beamforming factor $\frac{1}{N}\sum_{n=1}^{N}e^{j\theta_{n}}$ under discrete phase shift constraints can be approximated as $1-\frac{1}{6}{\left(\frac{\pi }{2^{q}}\right)}^{2}$. Consequently, the cascaded channel gain is scaled by the factor $1-\frac{1}{6}{\left(\frac{\pi }{2^{q}}\right)}^{2}$, and the term $\frac{1}{6}{\left(\frac{\pi }{2^{q}}\right)}^{2}$ represents the power loss due to phase quantization error. For higher quantization levels, the error diminishes and the scaling factor approaches unity, i.e., $\lim_{q\to \infty }\left(1-\frac{1}{6}{\left(\frac{\pi }{2^{q}}\right)}^{2}\right)=1$. Substituting this scaled channel gain into the end-to-end SNR expressions, the SOP under discrete phase shift constraints can be directly evaluated.

### 3.5. Incorporation of O-RAN Control Latency

In practical O-RAN deployments, the near-RT RIC executes xApps/rApps that compute RIS phase configurations based on periodically reported CSI measurements. The O-RAN control loop incurs latency from three main sources: (i) fronthaul transport delay between the O-RU and O-DU, (ii) RIC processing time for xApp/rApp execution, and (iii) CSI measurement and reporting intervals. Let $T_{d}$ denote the aggregate O-RAN control loop latency. During this interval, the wireless channel continues to evolve, causing the CSI available at the RIC to become partially outdated.

To model channel aging induced by O-RAN latency, we adopt the classical Jakes’ model. The temporal correlation between the true channel at the measurement instant and the channel at the time of RIS configuration application is given by $\rho_{T}=J_{0}\left(2\pi f_{d}T_{d}\right)$, where $f_{d}$ is the maximum Doppler frequency and $J_{0}\left(\cdot \right)$ is the zeroth-order Bessel function of the first kind. The aged channel coefficient $\tilde{h}$ relates to the estimated CSI $\hat{h}$ as

(44) $$\begin{array}{c} \tilde{h}=\rho_{T}\hat{h}+\sqrt{1-\rho_{T}^{2}}\;w, \end{array}$$

where w∼CN(0,1) is independent of $\hat{h}$.

Combining the estimation error model in (5), (19) and (20) with the aging model, the effective CSI estimation accuracy incorporating both estimation inaccuracy and O-RAN latency becomes $\rho_{\mathrm{eff}}=\rho \cdot \rho_{T}$. For notational convenience, we define the normalized latency $\delta_{d}=f_{d}T_{d}$, which jointly captures the Doppler spread and the RIC control loop delay. Substituting $\rho_{\mathrm{eff}}$ for ρ in the analytical expressions derived in Section 3 directly yields the secrecy performance metrics under the combined effects of imperfect CSI and O-RAN control latency. This unified treatment enables a systematic investigation of how RIC latency tolerance and CSI reporting periodicity impact PLS guarantees in O-RAN-enabled NTN architectures.

## 4. Numerical Results and Discussion

### 4.1. Numerical Results

This section examines the security performance of the RIS-assisted FSO/RF satellite downlinks through MC simulations with 10^6^ independent trials. To simplify the system analysis, AWGN with zero mean and equal variance is assumed to be present on all channels.

In the following experiments, the average SNR at U, denoted by ${\bar{\gamma }}_{2}^{u}$, is varied by adjusting the relay transmit power $P_{2}$ while keeping the noise power $\sigma_{u}^{2}$ fixed. Consequently, increasing ${\bar{\gamma }}_{2}^{u}$ genuinely represents an increase in the relay’s transmit power, and the high-SNR asymptotic regime corresponds to the practically relevant scenario of allocating higher power budgets at the relay. The FSO link average SNR ${\bar{\gamma }}_{1}$ is proportionally scaled with ${\bar{\gamma }}_{2}^{u}$ to maintain a realistic power relationship between the satellite and terrestrial segments. This ensures that the asymptotic limits derived in Section 3 reflect physically attainable operating conditions rather than mathematical artifacts.

The CDF presented in (6) contains an infinite sum, which is a direct consequence of employing the Maclaurin series expansion for the exponential function. To quantify the truncation error, we define the mean squared error (MSE) as

(45) $$\begin{array}{c} MSE=E\left\{{\left[{\bar{F}}_{\gamma_{1}}\left(\gamma \right)-{\hat{F}}_{\gamma_{1}}\left(\gamma \right)\right]}^{2}\right\}. \end{array}$$

where ${\bar{F}}_{\gamma_{1}}\left(\gamma \right)$ and ${\hat{F}}_{\gamma_{1}}\left(\gamma \right)$ denote the simulated CDF and the approximated CDF with *k* summation terms, respectively.

**Figure 2:**
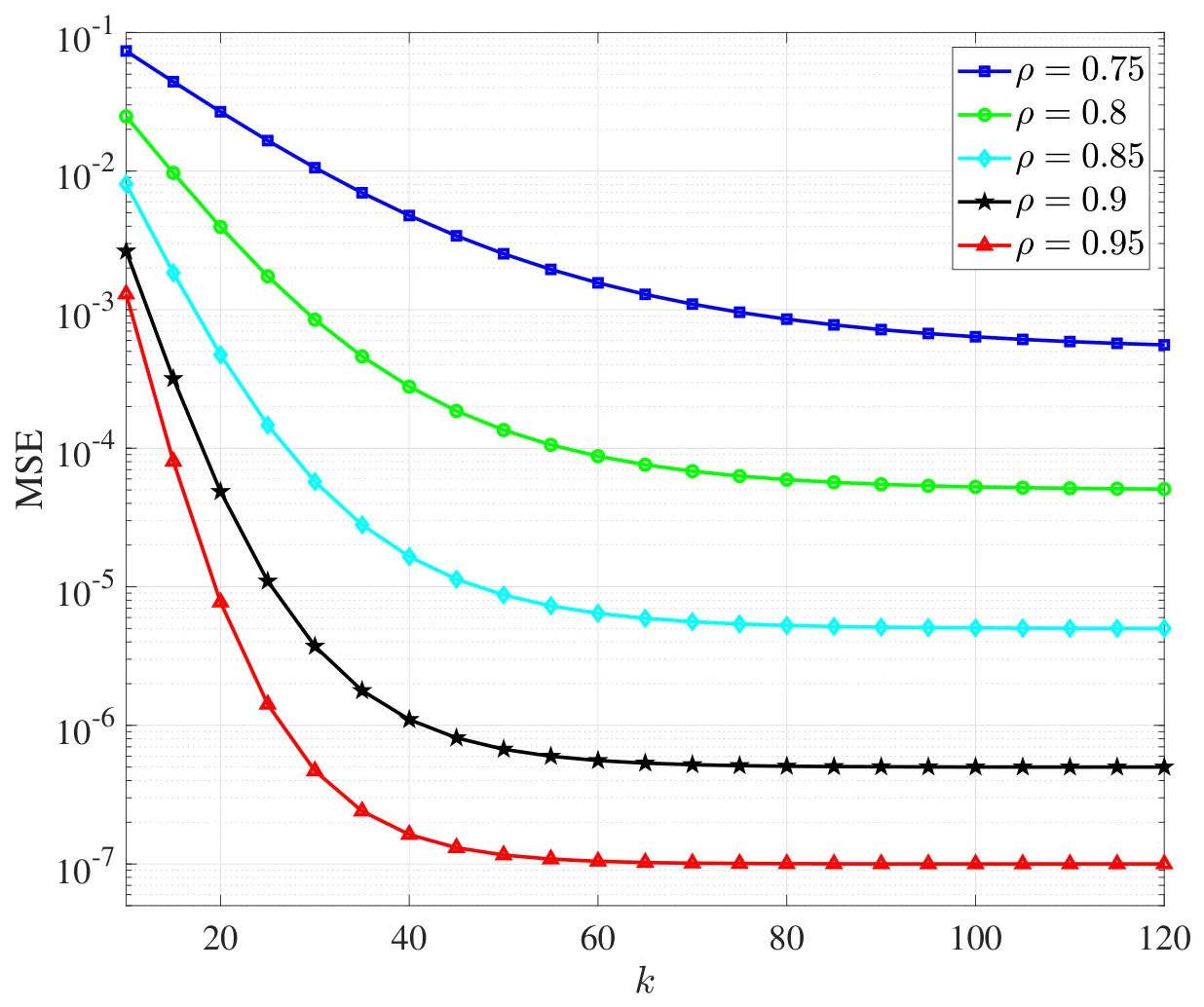
The relationship between MSE and *k* for different values of ρ.

Figure 2 illustrates the relationship between MSE and *k* for different values of ρ. It can be observed that for a given ρ, the infinite series converges to a finite truncation due to the properties of the exponential function. Moreover, a larger ρ leads to a faster convergence rate compared to a smaller ρ. To ensure accuracy while reducing computational time, we adopt a finite truncation at k=100 in place of the infinite summation. The other detailed parameters are provided in Table 2. Specifically, the parameters of the FSO link, including the atmospheric turbulence factors, the scatter and direct components, and the pointing error factors, are adopted from the M turbulence channel model characterized in [50]. The secure transmission threshold is set according to [35], and the deterministic phase values follow those used in [37]. In addition, the satellite-related parameters, including antenna gains, beam angles, and the FSO and RF link distances, are based on the satellite-terrestrial scenario in [36] and are consistent with the 3GPP TR 38.901 urban micro-cell channel model [63]. We note that the RIS-U and RIS-E links are modeled as independent Rayleigh fading channels, which is valid when U and E are separated by more than half a wavelength. When the terminals are in extremely close proximity, spatial correlation may arise between their channels, further degrading the secrecy performance; incorporating this effect is an important direction for future work.

**Figure 3:**
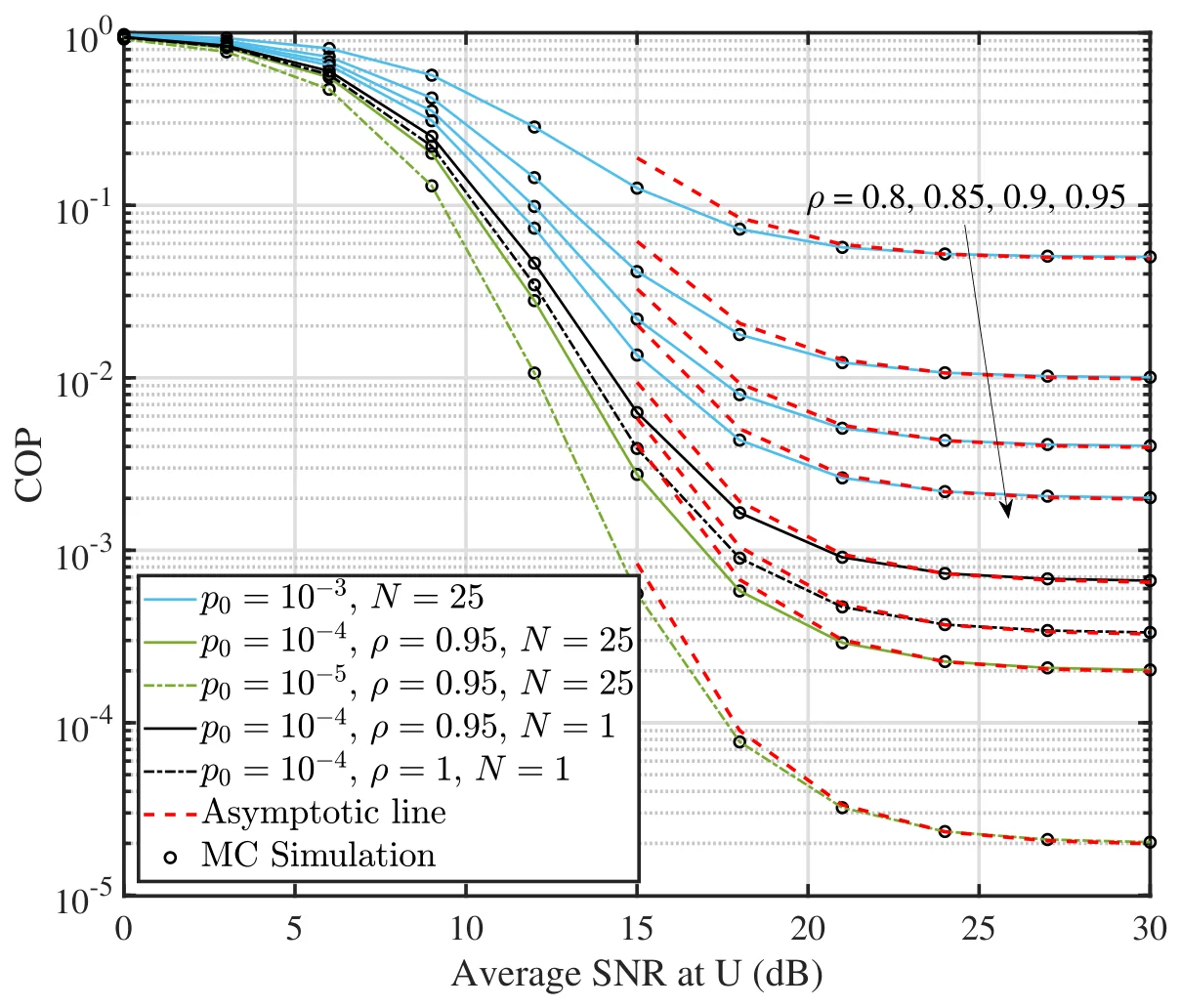
COP vs. average SNR at U with N=25 and N=1.

**Figure 4:**
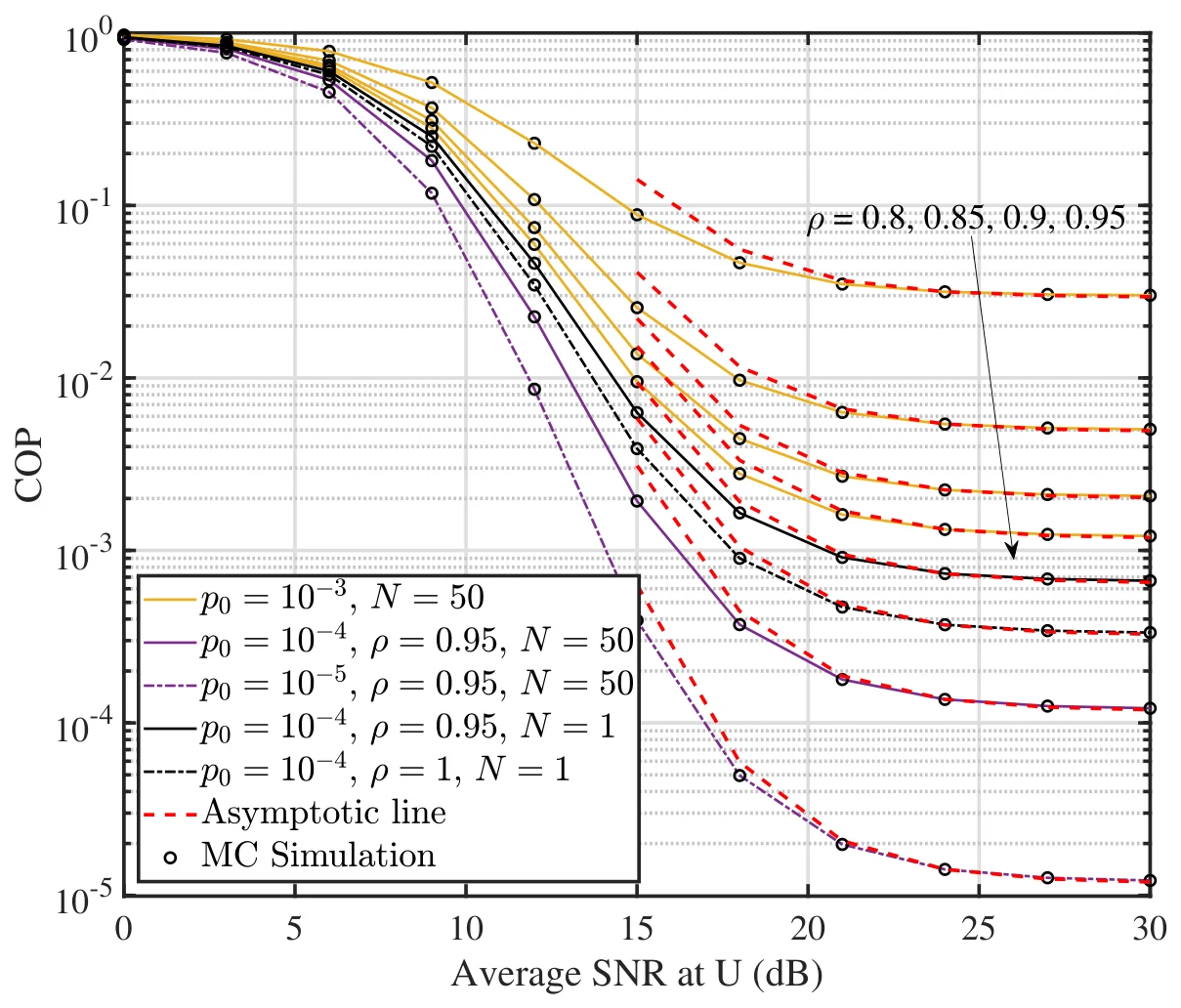
COP vs. average SNR at U with N=50 and N=1.

**Figure 5:**
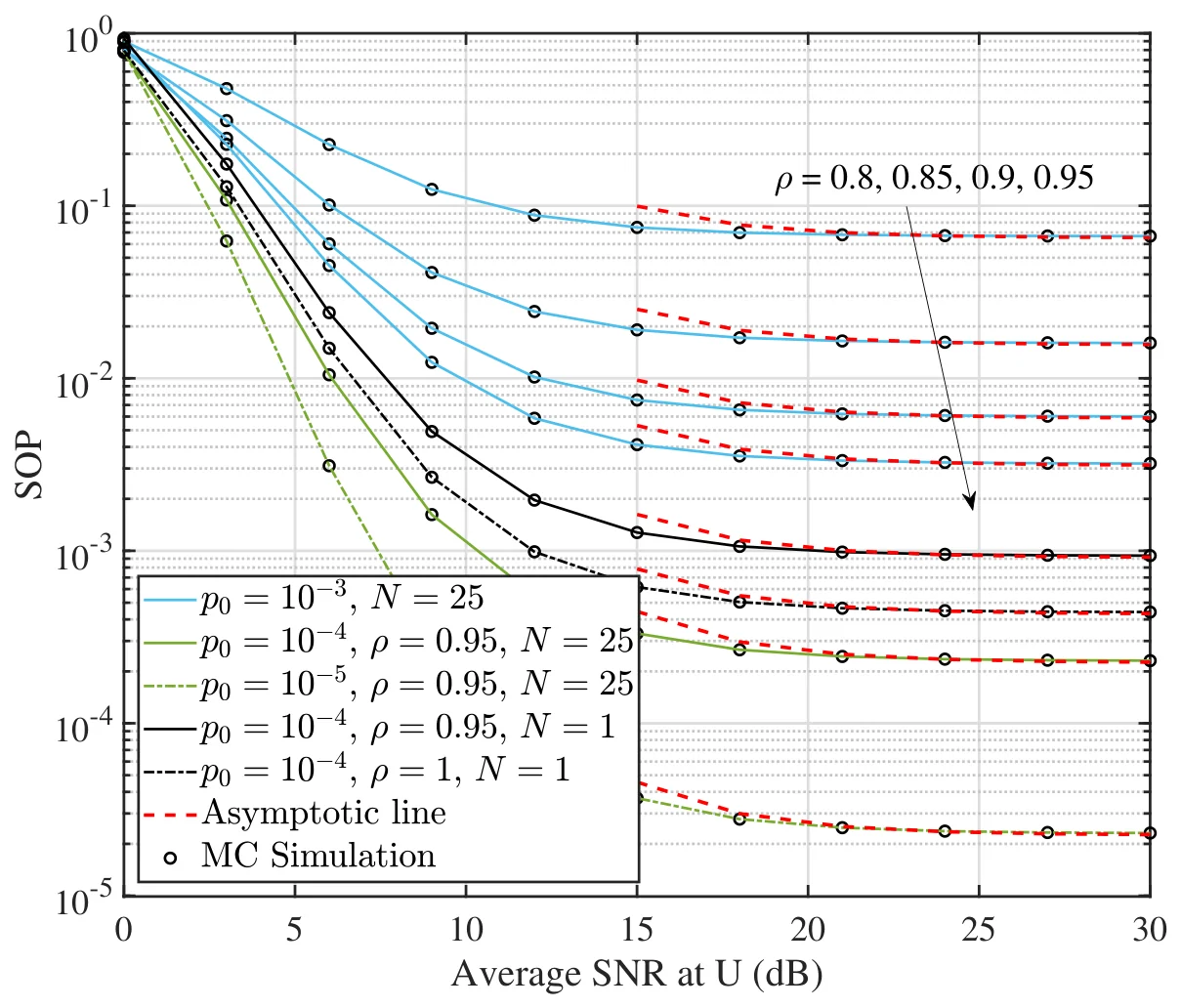
SOP vs. average SNR at U with N=25 and N=1.

**Figure 6:**
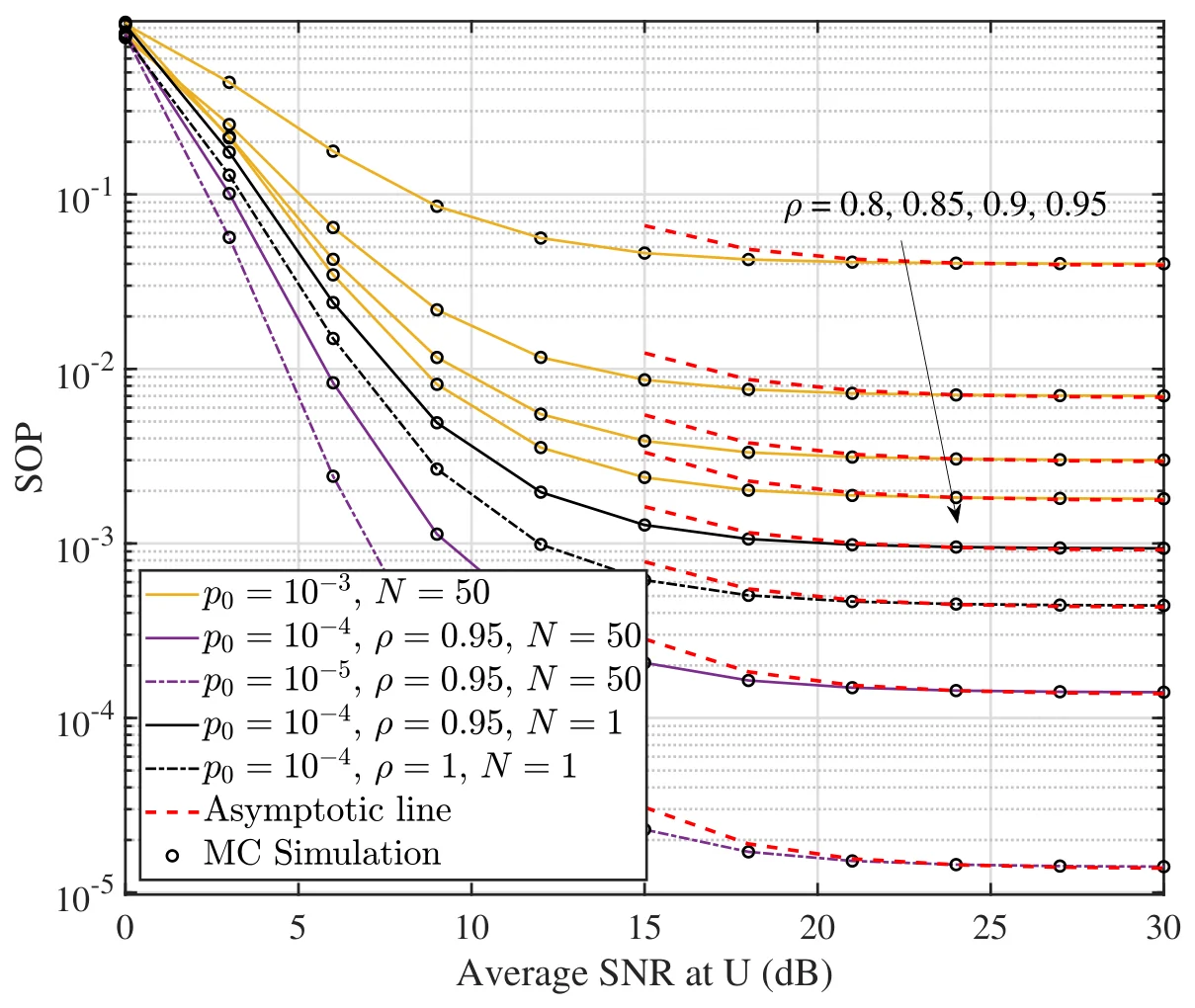
SOP vs. average SNR at U with N=50 and N=1.

Figure 3, Figure 4, Figure 5 and Figure 6 illustrate the fundamental trade-off between reliability and security through the behavior of COP and SOP as functions of the average SNR at U. The close agreement between MC simulations and analytical curves validates the proposed theoretical framework. Both metrics saturate under high-SNR regimes and the asymptotic analysis exhibits high accuracy. Three main insights are derived:

(i) Synergistic enhancement of link connectivity and confidentiality: Improving channel estimation accuracy (ρ ↑) or increasing the number of RIS elements (N ↑), while reducing link blockage probability ($p_{0}$ ↓), synergistically improves link connectivity (COP ↓) and confidentiality (SOP ↓).
(ii) Compensation for imperfect CSI: The second to fourth curves from the bottom in Figure 3, Figure 4, Figure 5 and Figure 6 demonstrate that RIS-assisted systems with N=25 or 50 elements can outperform a perfect-CSI baseline (ρ=1) with minimal RIS assistance (N=1). This highlights the capability of RIS to mitigate both channel uncertainty and limitations in eavesdropper channel knowledge.
(iii) SOP consistently exceeds COP: The SOP consistently exceeds the COP at the same average SNR levels. This results from the intrinsic relationship wherein connection outages inherently lead to secrecy outages, which constitutes a foundational constraint in the satellite downlink transmission system requiring simultaneous service continuity and confidentiality.

**Figure 7:**
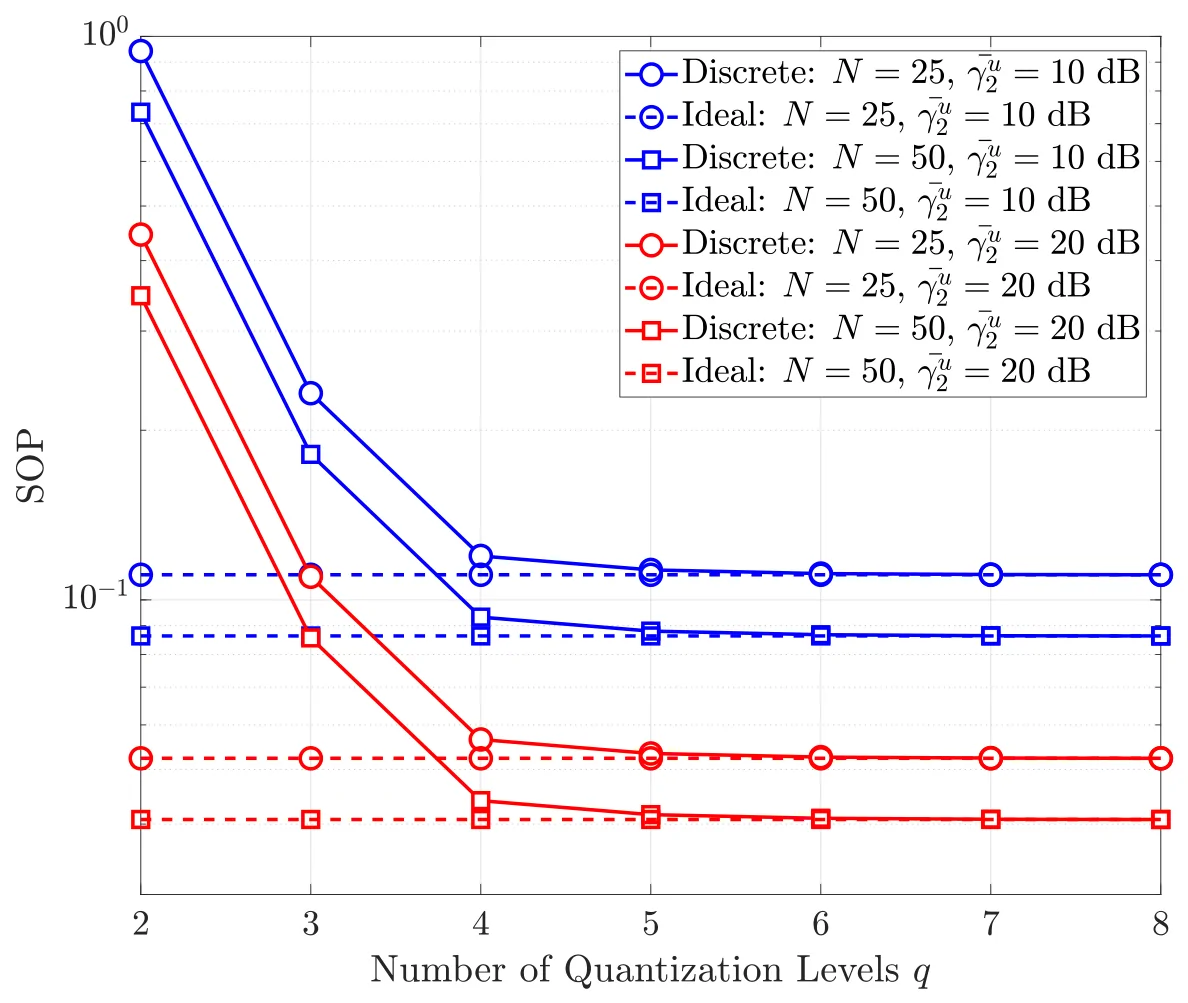
SOP vs. number of quantization levels *q*.

To provide a rigorous secrecy analysis under practical hardware constraints, we conduct simulations to evaluate the SOP versus the number of quantization bits *q*, as shown in Figure 7. Our simulation results verify that when the number of quantization bits is sufficiently high (e.g., q≥5, corresponding to $2^{q}\geq 32$ discrete levels), the SOP gap between the discrete phase shift case and the ideal continuous-phase benchmark becomes negligible. This confirms the robustness of our proposed framework, showing that high-precision secrecy can be maintained with finite-bit RIS hardware in practical satellite-terrestrial networks.

**Figure 8:**
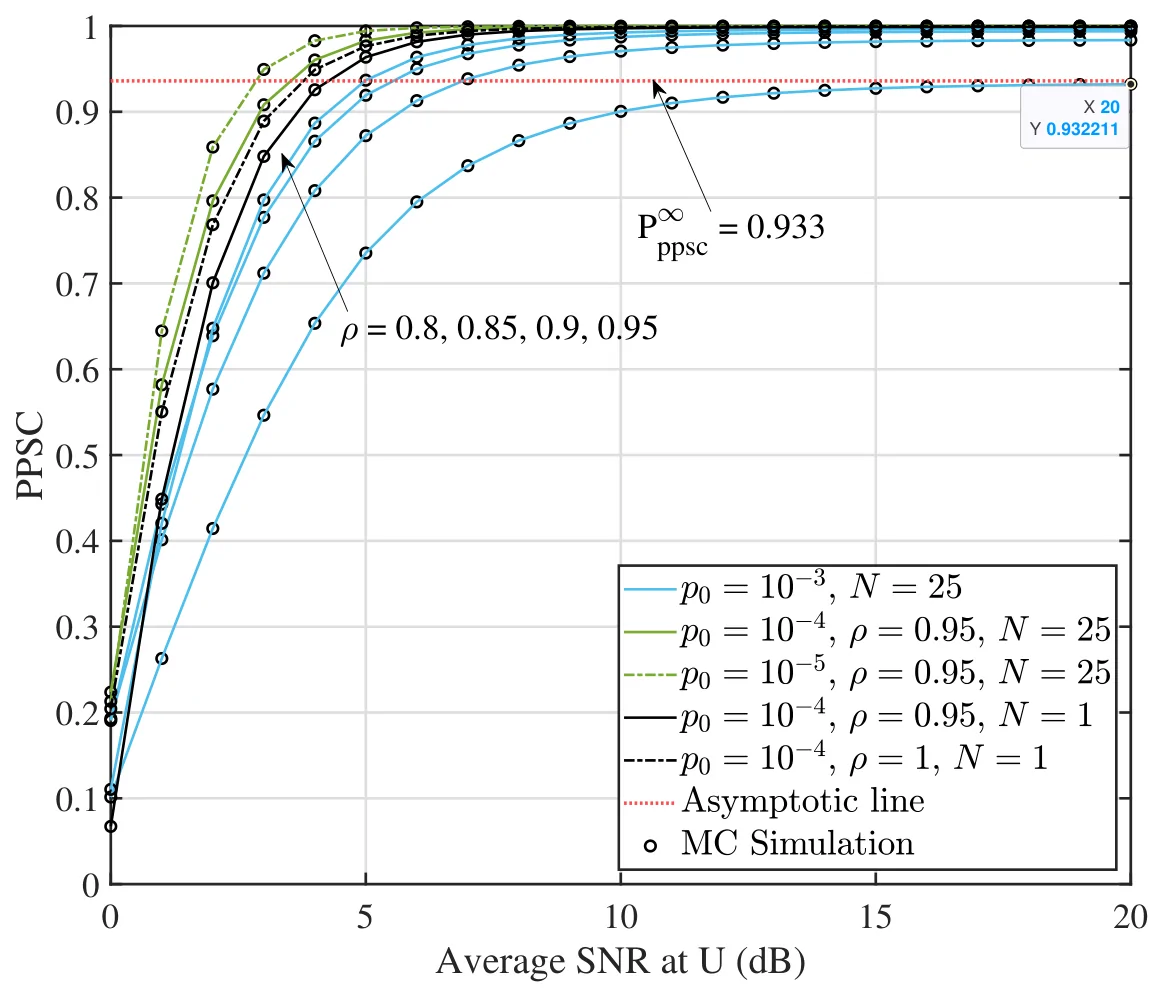
PPSC vs. average SNR at U with N=25 and N=1.

**Figure 9:**
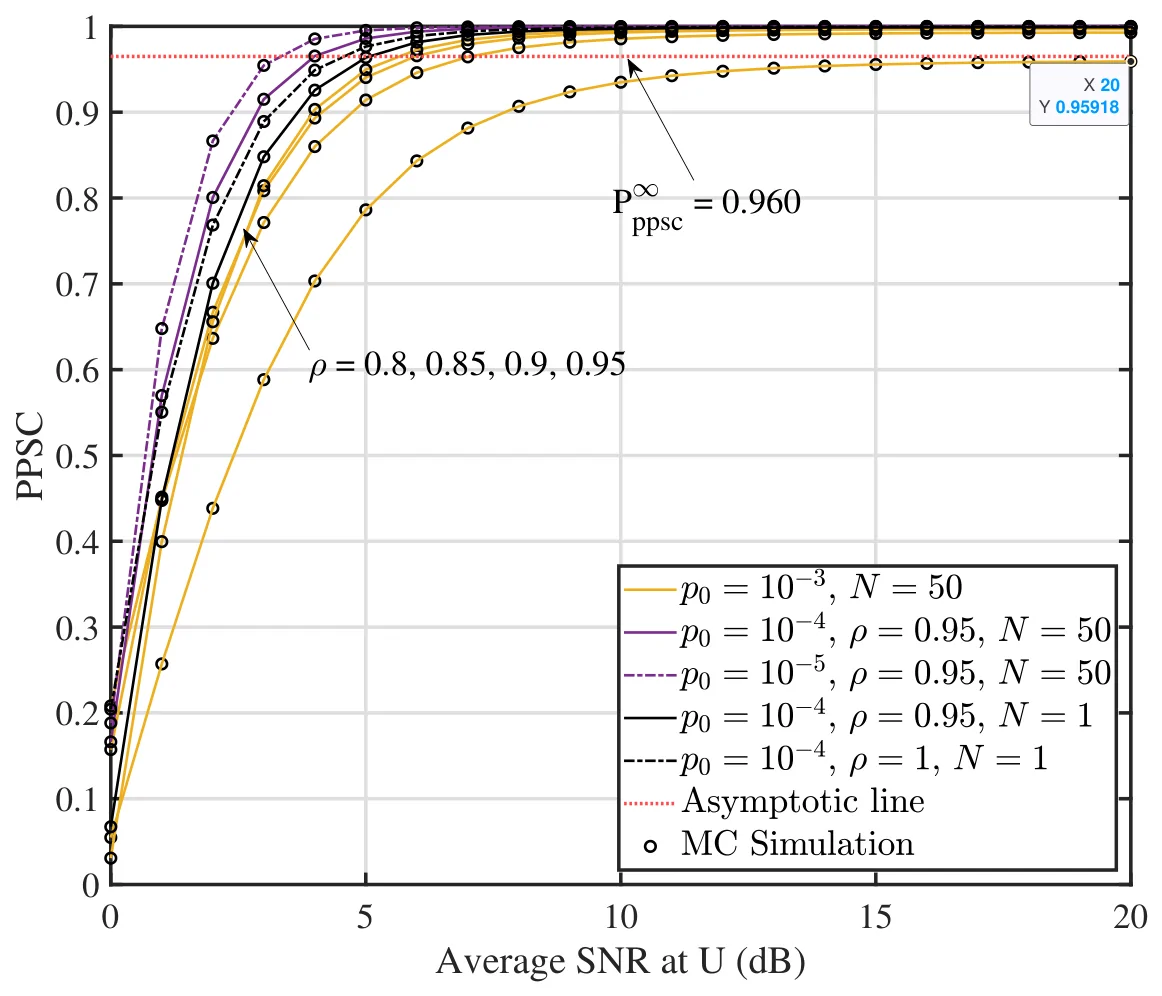
PPSC vs. average SNR at U with N=50 and N=1.

Figure 8 and Figure 9 quantify the operational confidentiality boundaries of the satellite downlink transmission system by examining the behavior of PPSC as a function of average SNR at U. Three key insights are observed:

(i) Security scalability with quantifiable design thresholds: The PPSC asymptotically approaches unity as ρ, *N* and the average SNR increase, while $p_{0}$ decreases. Specifically, our numerical results reveal that when $p_{0}\leq 10^{-3}$, N≥25, and ρ≥0.8, the system achieves PPSC >0.93 at SNR ≥20dB, representing a practical operating region for secure communications. This establishes clear design thresholds and highlights the system’s ability to achieve both wide-area coverage and physical-layer security through coordinated parameter optimization.
(ii) High-SNR model validation: At 20 dB average SNR, the close agreement between analytical and asymptotic PPSC values of 0.932 (analytical) versus 0.933 (asymptotic) and 0.959 (analytical) versus 0.960 (asymptotic) confirms the accuracy of the proposed model in capturing channel uncertainties.
(iii) Performance saturation near ideal confidentiality: The convergence of PPSC toward 1 indicates a fundamental security-capacity limit of the system, beyond which further increases in SNR yield diminishing improvements in confidentiality.

**Figure 10:**
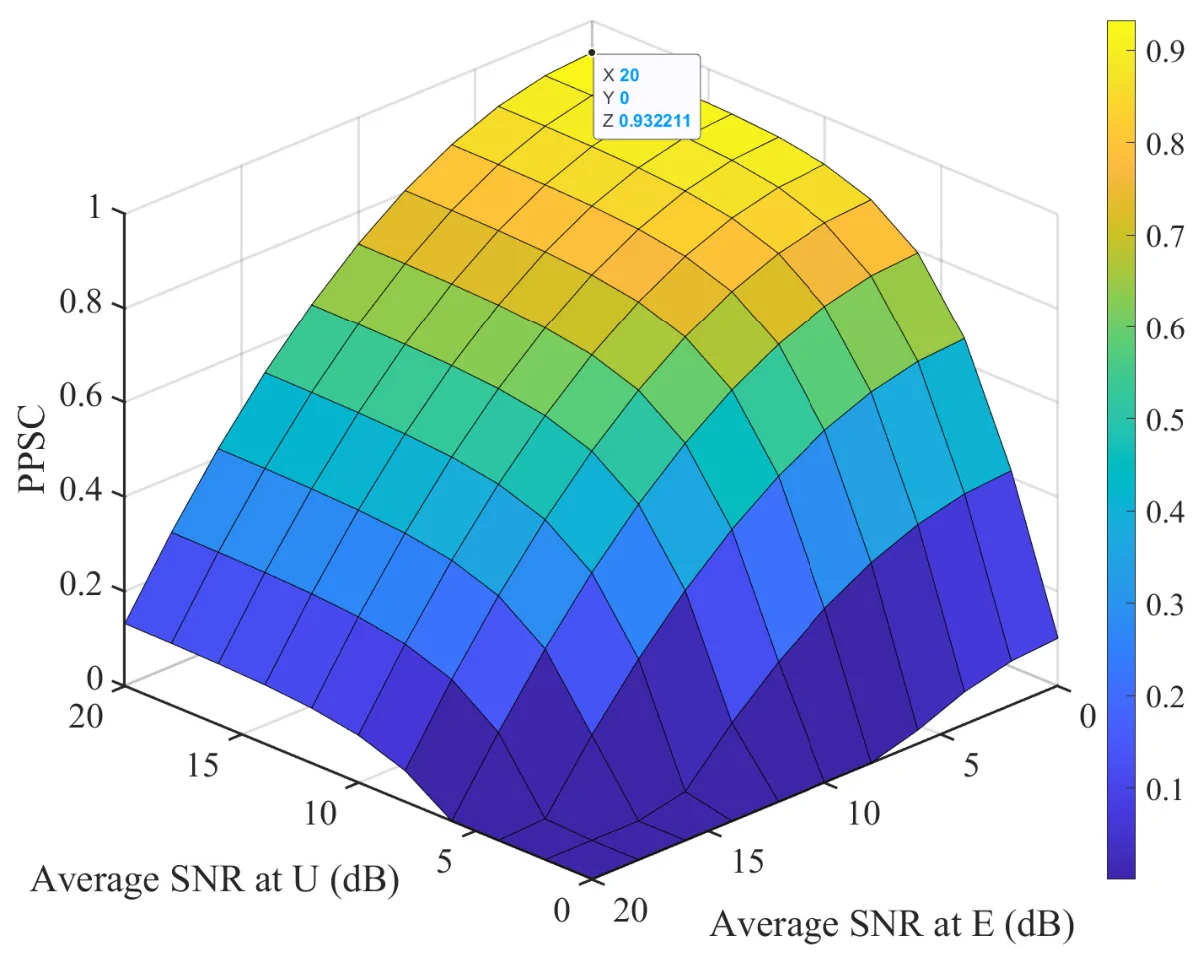
PPSC vs. average SNR at U/E with N=25, ρ=0.8 and $p_{0}=10^{-3}$.

Figure 10 shows the relationship between PPSC and the average SNR at U/E with N=25, ρ=0.8 and $p_{0}=10^{-3}$. As observed, when the eavesdropping SNR ${{\bar{\gamma }}_{2}}^{e}$ is 0 dB and the legitimate link SNR ${{\bar{\gamma }}_{2}}^{u}$ is 20 dB, the PPSC reaches 0.9322, which is consistent with the result shown in Figure 8. Moreover, a high eavesdropping SNR (${{\bar{\gamma }}_{2}}^{e}>10$ dB) severely degrades secrecy performance. This degradation is particularly acute when the legitimate link is weak (${{\bar{\gamma }}_{2}}^{u}=0$ dB), driving the PPSC below 0.1. To enhance secrecy, increasing the number of RIS elements and improving channel estimation accuracy can be adopted to strengthen the SNR advantage of the legitimate link, thereby improving overall system secrecy.

Figure 5 and Figure 6 reveal a fundamental security limitation: the link blockage probability $p_{0}$ and the CSI estimation accuracy ρ jointly govern the asymptotic secrecy performance. Under high-SNR regimes, the SOP approaches the asymptotic floor $F_{sop}\left(p_{0},\rho \right)=p_{0}+\left(1-p_{0}\right)\left[1-K\left(\rho \right)\right]$. This convergence occurs because, under high-SNR regimes, the constant terms in (37) dominate, while other terms diminish to negligible levels. This observation is consistent with the conclusion drawn in Remark 4. Consequently, $p_{0}$ and ρ are established as the joint fundamental bottlenecks for secrecy performance. Three insights emerge from our analysis:

(i) RIS-driven enhancement of link confidentiality: Increasing the number of RIS elements (N ↑) drives SOP toward its floor $F_{\mathrm{sop}}\left(p_{0},\rho \right)$. By concentrating signal energy precisely on the intended receiver and mitigating information leakage, RIS enables the system to approach its ultimate performance limit governed jointly by $p_{0}$ and ρ.
(ii) Improvement via CSI accuracy: A higher value of ρ leads to a smaller K(ρ) and consequently a lower SOP floor. This occurs because improving channel estimation accuracy (ρ ↑) provides more precise CSI, which fundamentally reduces the information leakage to eavesdroppers.
(iii) A three-dimensional system design paradigm: Achieving optimal secrecy performance entails balancing a critical trade-off among $p_{0}$ (environmental blockage risk), *N* (deployment costs), and ρ (channel feedback overhead). Our simulation results show that both environmental resilience (reducing $p_{0}$) and CSI quality (increasing ρ) must be jointly optimized. For satellite-terrestrial architectures with inherently limited CSI feedback, priority should be given to blockage mitigation while maintaining ρ above the critical threshold that prevents excessive elevation of the SOP floor.

### 4.2. Impact of O-RAN Control Latency

**Figure 11:**
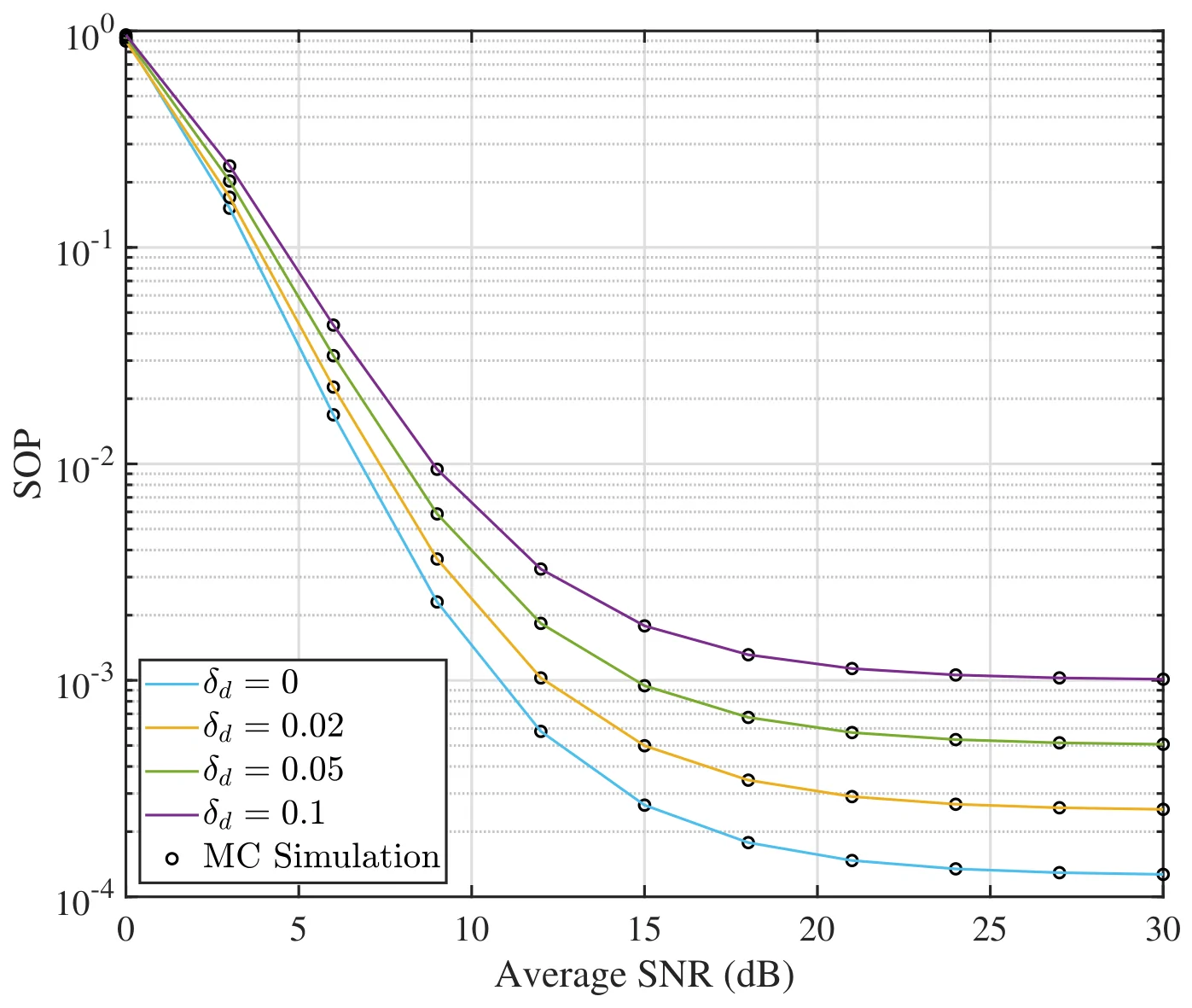
SOP vs. average SNR at U for different O-RAN latency levels ($\delta_{d}=0,\;0.02,\;0.05,\;0.1$) with N=50, ρ=0.95, and $p_{0}=10^{-4}$.

Figure 11 illustrates the impact of O-RAN control loop latency on the SOP performance. Four representative normalized latency levels are considered: $\delta_{d}=0$ (ideal, zero-latency RIC), $\delta_{d}=0.02$ (low latency, consistent with sub-10 ms RIC response and pedestrian mobility), $\delta_{d}=0.05$ (moderate latency, e.g., congested fronthaul or vehicular mobility), and $\delta_{d}=0.1$ (high latency, e.g., overloaded RIC or high-Doppler scenarios). As $\delta_{d}$ increases, the effective CSI accuracy $\rho_{\mathrm{eff}}=\rho \cdot \rho_{T}$ degrades, elevating SOP substantially. This result highlights that O-RAN control latency is not merely a QoS concern but a first-order security parameter: without adequate latency guarantees from the RIC and fronthaul, the PLS gains promised by RIS-assisted architectures can be largely negated by channel aging. Practical countermeasures include deploying hardware-accelerated RIC platforms, optimizing xApp/rApp execution pipelines, and implementing channel prediction algorithms at the RIC to compensate for aging during the control loop latency.

### 4.3. Design Insights for Practical System Deployment

The analytical and asymptotic results derived in this work provide several practical guidelines for designing secure RIS-assisted mixed FSO/RF satellite downlink systems within O-RAN-enabled NTN architectures.

- RIS element configuration: The asymptotic SOP expression indicates that increasing the number of RIS elements *N* improves the secrecy performance until the system becomes blockage-limited. Beyond this point, additional RIS elements yield diminishing returns. This provides a quantitative basis for determining the minimum *N* required to meet a target SOP under given channel conditions, thereby avoiding unnecessary hardware costs.
- CSI accuracy requirements:The SOP expressions incorporate the CSI correlation coefficient ρ, enabling designers to determine the minimum estimation accuracy required for a target security level. In practice, ρ can be improved by increasing pilot length or employing advanced estimation algorithms (such as sparse Bayesian learning [45]), and maintained above the critical threshold by adapting the pilot overhead to the channel coherence time.
- Blockage mitigation: Random link blockage and imperfect CSI jointly establish a fundamental SOP lower bound that cannot be overcome by increasing the SNR. In real deployments, $p_{0}$ can be reduced through careful RIS site selection (e.g., elevated locations with clear line-of-sight) or by deploying multiple spatially separated RIS panels to achieve macroscopic diversity against blockages.
- Power allocation: The PPSC analysis reveals that once the system enters the blockage-limited regime, further increasing the transmit power does not yield proportional secrecy gains. This insight guides energy-efficient relay power allocation, which is critical for satellite systems with stringent power budgets. The derived expressions also enable joint optimization of the target secrecy rate $R_{th}$ and the RIS configuration to balance connectivity and confidentiality according to application-specific priorities.
- O-RAN integration and latency awareness: The proposed secrecy analysis framework can be naturally integrated into the O-RAN architecture by deploying the COP/SOP/PPSC analytical models as xApps within the near-RT RIC. These xApps can leverage real-time channel measurements collected through O-RAN’s open E2 interface to dynamically compute the optimal RIS phase configuration, relay gain, and secure transmission rate $R_{th}$, enabling closed-loop security optimization across heterogeneous satellite-terrestrial segments. Our latency analysis in Section 3.5 further reveals that O-RAN control loop latency is a first-order security parameter, and the derived $\rho_{\mathrm{eff}}=\rho \cdot \rho_{T}$ model provides a quantitative tool for specifying the maximum tolerable RIC latency and CSI reporting periodicity to meet target SOP requirements.

## 5. Conclusions

In this paper, we conduct a secrecy analysis of a novel RIS-assisted mixed FSO/RF satellite downlink transmission system within an O-RAN-enabled NTN framework, accounting for imperfect CSI and link blockage. The FSO link is modeled by the M distribution, while the RF link is assumed to undergo Rayleigh fading aided by RIS. We derive the closed-form expressions for three critical performance metrics, namely COP, SOP, and PPSC, as well as conduct the asymptotic analysis to uncover the system’s fundamental performance limits. We also incorporate the practical constraint of discrete phase shifts at the RIS. Numerical results demonstrate that link blockage probability and channel estimation accuracy jointly govern the secrecy performance floor. Moreover, increasing the number of RIS elements and improving the accuracy of channel estimation synergistically reduce eavesdropping vulnerabilities inherent in open wireless transmission. These findings offer practical insights for system operators to balance connectivity and confidentiality in O-RAN-enabled next-generation satellite-terrestrial infrastructures, with particular relevance for emergency communications and mobile satellite services susceptible to dynamic blockages. Future work will explore AI/ML-driven RIS beamforming optimization within O-RAN’s open control framework, latency-aware xApp design that accounts for the channel aging effects quantified in this work, and adaptive blockage mitigation to enhance the resilience of global satellite communication networks.

## Figures and Tables

**Table 1:** Contrasting our work with the state of the art.

Ref.	Secrecy Analysis	RIS-Assisted	FSO	RF	CSI	Blockage
[27]	✕	✕	Gamma-Gamma	Rayleigh	perfect	✕
[28]	✕	✓	EG-distribution	$K_{G}$ fading	perfect	✕
[29]	✓	✕	Gamma-Gamma	Elev. angle-dep.	perfect	✕
[30]	✓	✕	Gamma-Gamma	Nakagami-*m*	perfect	✕
[31]	✓	✕	M distribution	η-μ fading	perfect	✕
[32]	✓	✕	M distribution	Nakagami-*m*	perfect	✕
[33]	✓	✕	Geometric loss	Nakagami-*m*	perfect	✕
[34]	✓	✓	Shadowed-Rician	Rayleigh	perfect	✕
[35]	✓	✓	Gamma-Gamma	Nakagami-*m*	perfect	✕
[36]	✕	✓	M distribution	Rayleigh	imperfect	✕
[37]	✓	✕	M distribution	Nakagami-*m*	imperfect	✕
[38]	✕	✓	Gamma-Gamma	Heterogeneous	imperfect	✕
Our work	✓	✓	M distribution	Rayleigh	imperfect	✓

**Table 2:** Simulation parameters.

Parameter	Symbol	Value
Atmospheric turbulence factor	$\left[a,\;b\right]$	[3, 4.2]
Scatter and direct components	$\left[g,\;2b_{0},\;\chi \right]$	[0.15, 0.05, 1.25]
Pointing error factor	$\left[A_{0},\;\psi \right]$	[0.8, 1.1]
Distance of FSO link	$d_{s}$	35,786 km
Distance of RF link	$\left[d_{r},\;d_{u},\;d_{e}\right]$	[1.5, 0.5, 0.5] km
Satellite beam gain	$G_{a}$	52 dB
Receive antenna gain	$G_{b}$	5 dB
Optical signal wavelength	λ	1550 nm
Beam angles	$\left[\varphi ,\;\varphi_{3\mathrm{dB}}\right]$	[0.01°, 0.4°]
Channel loss of RF link	$C_{0}$	−98.5 dB
Path loss factor	τ	3
Optical–electrical conversion	η	0.8
Channel attenuation loss	$h_{a}$	−4.8 dB
Deterministic phases	$\varphi_{A}-\varphi_{B}$	π/2
Detection method	*t*	1 (HD)
Average SNR at E	${{\bar{\gamma }}_{2}}^{e}$	0 dB
Fixed relay gain	Υ	1.5
Secure transmission threshold	$R_{th}$	0.01 bps

## Data Availability

The original contributions presented in this study are included in the article. Further inquiries can be directed to the corresponding author.

## Appendix A. Derivation of Connection Outage Probability Expression

To derive the expression for COP, the provided CDF and PDF need to be substituted into the original integral, as follows:

(A1) $$P_{cop}\left(\gamma_{th}\right)=F_{\gamma_{1}}\left(\gamma_{th}\right)+\underset{I_{1}}{\underset{︸}{\int_{0}^{\infty }F_{\gamma_{2}^{u}}\left(\frac{\Upsilon \gamma_{th}}{\omega }\right)f_{\gamma_{1}}\left(\gamma_{th}+\omega \right)d\omega }}.$$

First, decompose $F_{\gamma_{2}^{u}}\left(\frac{\Upsilon \gamma_{th}}{\omega }\right)$ into

(A2) $$F_{\gamma_{2}^{u}}\left(\frac{\Upsilon \gamma_{th}}{\omega }\right)=p_{0}+\hat{p_{0}}{\hat{F}}_{\gamma_{2}^{u}}\left(\frac{\Upsilon \gamma_{th}}{\omega }\right),$$

where $\hat{p_{0}}=1-p_{0}$ and

(A3) $${\hat{F}}_{\gamma_{2}^{u}}\left(\frac{\Upsilon \gamma_{th}}{\omega }\right)=\frac{G_{2,3}^{2,2}\left[\left.\frac{\Lambda^{2}\Upsilon \gamma_{th}}{\rho^{2}u_{u}\omega }\right|\begin{array}{c} 1-\nu_{u},1\\ m_{1},m_{2},0 \end{array}\right]}{\Gamma \left(m_{1}\right)\Gamma \left(m_{2}\right)\Gamma \left(\nu_{u}\right)}.$$

Subsequently, we can obtain the expression for $I_{1}$

(A4) $$\begin{array}{cc} I_{1} & =\int_{0}^{\infty }\left[p_{0}+\hat{p_{0}}{\hat{F}}_{\gamma_{2}^{u}}\left(\frac{\Upsilon \gamma_{th}}{\omega }\right)\right]f_{\gamma_{1}}\left(\gamma_{th}+\omega \right)d\omega \\ \; & =p_{0}\left[1-F_{\gamma_{1}}\left(\gamma_{th}\right)\right]+\hat{p_{0}}I_{2}, \end{array}$$

where $I_{2}=\int_{0}^{\infty }{\hat{F}}_{\gamma_{2}^{u}}\left(\frac{\Upsilon \gamma_{th}}{\omega }\right)f_{\gamma_{1}}\left(\gamma_{th}+\omega \right)d\omega$. Using the Mellin–Barnes form of the Meijer-*G* function [59] (Equation (9.301)), we can obtain the expression for $I_{2}$ as

(A5) $$\begin{array}{cc} I_{2} & =\sum_{i=1}^{a}\sum_{k=0}^{\infty }\frac{A_{1}b_{i}\Delta }{\Gamma \left(m_{1}\right)\Gamma \left(m_{2}\right)\Gamma \left(\nu_{u}\right)}\frac{1}{{\left(2\pi j\right)}^{2}}\int_{L_{1}}\int_{L_{2}}\phi \left(s_{1}\right)\Gamma \left(s_{2}\right)\Omega^{-s_{2}}\\ \; & \;\times {\left(\frac{\Lambda^{2}\Upsilon \gamma_{th}}{\rho^{2}u_{u}\omega }\right)}^{s_{1}}\int_{0}^{\infty }{\left(\gamma_{th}+\omega \right)}^{\kappa }\omega^{-s_{1}}d\omega \;ds_{2}\;ds_{1}. \end{array}$$

where $L_{1}$ and $L_{2}$ are the boundaries in the $s_{1}$-plane and $s_{2}$-plane, respectively; $\phi \left(s_{1}\right)=\frac{\Gamma \left(m_{1}-s_{1}\right)\;\Gamma \left(m_{2}-s_{1}\right)\;\Gamma \left(\nu_{u}+s_{1}\right)\;\Gamma \left(s_{1}\right)}{\Gamma (1+s_{1})}$ and $\kappa =\frac{k+1-2s_{2}}{t}-1$.

Employing Mellin–Barnes contour integration techniques [60] (Equation (1.1)), we rigorously establish the extended generalized bivariate *H* function representation in (31) through systematic analytical derivation, thereby formally concluding the proof.

## Appendix B. Derivation of Asymptotic Connection Outage Probability Expression

The asymptotic COP under high-SNR conditions is derived through rigorous application of Mellin–Barnes contour integration and Meijer-*G* function approximations. Beginning with the integral representation in (32),

(A6) $$\begin{array}{cc} P_{cop}^{\infty } & =F_{\gamma_{1}}^{\infty }\left(\gamma_{th}\right)+\int_{0}^{\infty }F_{\gamma_{2}^{u}}^{\infty }\left(\frac{\Upsilon \gamma_{th}}{\omega }\right)f_{\gamma_{1}}^{\infty }\left(\gamma_{th}+\omega \right)d\omega \\ \; & =p_{0}+\hat{p_{0}}F_{\gamma_{1}}^{\infty }\left(\gamma_{th}\right)+\hat{p_{0}}\underset{I_{3}}{\underset{︸}{\int_{0}^{\infty }{\hat{F}}_{\gamma_{2}^{u}}^{\infty }\left(\frac{\Upsilon \gamma_{th}}{\omega }\right)f_{\gamma_{1}}^{\infty }\left(\gamma_{th}+\omega \right)d\omega }}. \end{array}$$

First, we substitute the asymptotic CDF $F_{\gamma_{1}}^{\infty }\left(\gamma_{th}\right)=1-K$ into (A6), where K is defined in (14). Using the definition of the H-function referenced in [61] (Equation (1.1.2)), we can obtain the expression of $I_{3}$ as follows:

(A7) $$\begin{array}{cc} I_{3} & =\sum_{i=1}^{a}\sum_{k=0}^{\infty }\frac{A_{1}b_{i}\Delta }{\Gamma \left(m_{1}\right)\Gamma \left(m_{2}\right)\Gamma \left(\nu_{u}\right)}\frac{1}{2\pi j}\\ \; & \;\times \int_{L_{1}}\phi \left(s_{1}\right)\Omega^{-s_{2}}{\left(\frac{\Lambda^{2}\Upsilon \gamma_{th}}{\rho^{2}u_{u}\omega }\right)}^{s_{1}}\\ \; & \;\times H_{2,3}^{2,2}\left[\left.\Omega \gamma_{th}^{\frac{2}{t}}\right|\begin{array}{c} \left(1-s_{1},\frac{2}{t}\right),\left(1,1\right)\\ \left(\frac{1+k}{2},1\right),\left(1,\frac{2}{t}\right),\left(0,1\right) \end{array}\right]ds_{1}. \end{array}$$

Invoking the asymptotic formula [61] (Equation (1.5.9), Equation (1.8.4)), the Fox-*H* function estimates are as follows:

(A8) $$\begin{array}{cc} H_{2,3}^{2,2}\left[\left.\Omega \gamma_{th}^{\frac{2}{t}}\right|\begin{array}{c} \left(1-s_{1},\frac{2}{t}\right),\left(1,1\right)\\ \left(\frac{1+k}{2},1\right),\left(1,\frac{2}{t}\right),\left(0,1\right) \end{array}\right]\; & =\Gamma \left(\frac{1+k}{2}-\frac{t}{2}\right){\left(\Omega \gamma_{th}^{\frac{2}{t}}\right)}^{\frac{t}{2}}\Gamma \left(1+s_{1}\right)\\ \; & +\frac{2\Gamma \left(1-\frac{1+k}{t}\right)}{1+k}{\left(\Omega \gamma_{th}^{\frac{2}{t}}\right)}^{\frac{1+k}{2}}\Gamma \left(\frac{1+k}{t}+s_{1}\right). \end{array}$$

Upon substitution into the expression for $I_{3}$ and subsequent algebraic manipulation, we derive (33), thus concluding the proof.

## Appendix C. Derivation of Secrecy Outage Probability Expression

The SOP is defined in (35). Substituting the probability density function $f_{\gamma_{2}^{e}}\left(\gamma \right)$ from (22), we can split the integral into two parts: $P_{sop}=I_{4}+I_{5}$, where

(A9) $$\begin{array}{c} I_{4}=\int_{0}^{\infty }p_{0}P_{cop}\left(\gamma \varrho \right)\delta \left(\gamma \right)d\gamma , \end{array}$$

and

(A10) $$\begin{array}{cc} I_{5} & =\int_{0}^{\infty }\frac{\left(1-p_{0}\right)P_{cop}\left(\gamma \varrho \right)}{\Gamma \left(m_{1}\right)\Gamma \left(m_{2}\right)\Gamma \left(\nu_{e}\right)\gamma }G_{1,2}^{2,1}\left(\frac{\Lambda^{2}\gamma }{\rho^{2}u_{e}}|\begin{array}{c} 1-\nu_{e}\\ m_{1},m_{2} \end{array}\right)d\gamma . \end{array}$$

Using the sifting property of the Dirac delta function, we can obtain: $I_{4}=p_{0}P_{cop}\left(0\right)$. From the definition of the COP and the relationship given in (6) and (23), we have

(A11) $$\begin{array}{c} P_{cop}\left(0\right)=F_{\gamma_{1}}\left(0\right)+\int_{0}^{\infty }F_{\gamma_{2}^{u}}\left(0\right)f_{\gamma_{1}}\left(\omega \right)d\omega , \end{array}$$

where $F_{\gamma_{1}}\left(0\right)=K$ and $F_{\gamma_{2}^{u}}\left(0\right)=p_{0}$. Thus, we can get $I_{4}=p_{0}\left(K+p_{0}\right)$.

Substituting the expression for $P_{cop}\left(\gamma \varrho \right)$ from (31) into the integral for $I_{5}$,

(A12) $$\begin{array}{c} I_{5}=\frac{\left(1-p_{0}\right)}{\Gamma \left(m_{1}\right)\Gamma \left(m_{2}\right)\Gamma \left(\nu_{e}\right)}\left[p_{0}I_{A}+\left(1-p_{0}\right)I_{B}+\left(1-p_{0}\right)I_{C}\right], \end{array}$$

where $I_{A}$ corresponds to the constant term $p_{0}$ in $P_{cop}$ integrated with the continuous part of $f_{\gamma_{2}^{e}}$; $I_{B}$ corresponds to the Meijer-*G* function term in $P_{cop}$; $I_{C}$ corresponds to the extended generalized bivariate *H* function term in $P_{cop}$.

Using the normalization condition of the PDF,

(A13) $$\begin{array}{c} \int_{0}^{\infty }f_{\gamma_{2}^{e}}\left(\gamma \right)d\gamma =p_{0}+\frac{1-p_{0}}{\Gamma \left(m_{1}\right)\Gamma \left(m_{2}\right)\Gamma \left(\nu_{e}\right)}I_{A}=1, \end{array}$$

we obtain: $I_{A}=\Gamma \left(m_{1}\right)\Gamma \left(m_{2}\right)\Gamma \left(\nu_{e}\right)$. Therefore, we can further update the expression of $I_{5}$ to

(A14) $$\begin{array}{c} I_{5}=p_{0}\left(1-p_{0}\right)+\frac{{\left(1-p_{0}\right)}^{2}}{\Gamma \left(m_{1}\right)\Gamma \left(m_{2}\right)\Gamma \left(\nu_{e}\right)}\left(I_{B}+I_{C}\right). \end{array}$$

$I_{B}$ involves the integral of the product of two Meijer-*G* functions. Using the integral formula for the product of Meijer-*G* functions (involving Mellin transforms), this integral can be converted into a univariate Fox-*H* function (a generalization of the Meijer-*G* function), ultimately yielding the $H_{2,3}^{2,2}$ form, as shown in the second term of the target equation.

$I_{C}$ involves the integral of a bivariate Fox-*H* function with a Meijer-*G* function. Using the Mellin representation of Fox-*H* functions and the convolution theorem, this integral can be transformed into an extended generalized bivariate *H* function $H_{1,0;1,3;3,2}^{0,1;2,1;2,2}$ as shown in the third term of the target equation.

Adding $I_{4}$ and $I_{5}$, and substituting the results for $I_{A}$, $I_{B}$, $I_{C}$, we obtain

(A15) $$\begin{array}{c} P_{sop}=K_{p_{0}}+\frac{{\left(1-p_{0}\right)}^{2}}{\Gamma \left(m_{1}\right)\Gamma \left(m_{2}\right)\Gamma \left(\nu_{e}\right)}\left(I_{B}+I_{C}\right), \end{array}$$

where the constant term $K_{p_{0}}=p_{0}+K\left(1-p_{0}\right)p_{0}$, after algebraic combination, matches the definition in the main text.

Thus, we arrive at the closed-form expression for the secrecy outage probability given in (35) of the main text.

## Appendix D. Derivation of Asymptotic Secrecy Outage Probability Expression

We begin by defining $I_{6}$ as the extended generalized bivariate *H* function term appearing in the SOP expression in Equation (35). To derive the asymptotic expression for $P_{sop}^{\infty }$, we first express $I_{6}$ in its Mellin–Barnes double integral form. Applying the definition of the bivariate Fox-*H* function [59] (Equation (3.381.4)), we obtain the integral representation as

(A16) $$\begin{array}{cc} I_{6} & ={\left(\frac{1}{2\pi j}\right)}^{2}\int_{L_{1}}\int_{L_{2}}\frac{\Gamma \left(\frac{1+k}{2}+s_{1}\right)\Gamma \left(-s_{1}\right)\Gamma \left(1+\frac{2}{t}s_{1}\right)}{\Gamma \left(1-s_{1}\right)}\\ \; & \times \frac{\Gamma \left(m_{1}+s_{2}\right)\Gamma \left(m_{2}+s_{2}\right)\Gamma \left(\nu_{u}-s_{2}\right)}{\Gamma \left(1+s_{2}\right)}\Gamma \left(s_{2}-\frac{2}{t}s_{1}\right)\\ \; & \times {\left(\Omega \varrho \Upsilon^{\frac{2}{t}}\right)}^{-s_{1}}{\left(\frac{\Lambda^{2}\varrho }{\rho^{2}u_{u}u_{e}}\right)}^{-s_{2}}ds_{1}ds_{2}. \end{array}$$

The next step involves determining the dominant poles of the integrand under high-SNR regimes. The term ${\left(\Omega \varrho \Upsilon^{\frac{2}{t}}\right)}^{-s_{1}}$ plays the decisive role in the asymptotic analysis. As ${\bar{\gamma }}_{1}$ and ${\bar{\gamma }}_{2}^{u}\to \infty$, the parameter Ω→0, which dictates that we consider poles with the largest real parts for $s_{1}$.

We now apply the residue theorem to evaluate the double integral in the asymptotic limit. The dominant contributions come from the poles of $\Gamma \left(\frac{1+k}{2}+s_{1}\right)$ and other Gamma functions in the numerator. The asymptotic expansion is obtained by summing the residues at these dominant poles. For the dominant pole at $s_{1}=-\frac{1+k}{2}$, we obtain the term involving $G_{2,4}^{3,2}$, while the second dominant pole at another critical point yields the $G_{2,2}^{2,2}$ term. This process effectively reduces the extended generalized bivariate *H* function to a sum of univariate Meijer-*G* functions, which is characteristic of asymptotic analysis in multivariate special functions.

By employing [60] (Equation (1.1)) and [61] (Equation (1.8.4)), we derive the final asymptotic expression for $P_{sop}^{\infty }$ shown in (37). This completes the proof of the asymptotic secrecy outage probability expression.
